# FOXO3 Regulates CD8 T Cell Memory by T Cell-Intrinsic Mechanisms

**DOI:** 10.1371/journal.ppat.1002533

**Published:** 2012-02-16

**Authors:** Jeremy A. Sullivan, Eui Ho Kim, Erin H. Plisch, Stanford L. Peng, M. Suresh

**Affiliations:** 1 Department of Pathobiological Sciences, University of Wisconsin-Madison, Madison, Wisconsin, United States of America; 2 Virginia Mason Medical Center and Benaroya Research Institute, Seattle, Washington, United States of America; Nationwide Children's Hospital, United States of America

## Abstract

CD8 T cell responses have three phases: expansion, contraction, and memory. Dynamic alterations in proliferation and apoptotic rates control CD8 T cell numbers at each phase, which in turn dictate the magnitude of CD8 T cell memory. Identification of signaling pathways that control CD8 T cell memory is incomplete. The PI3K/Akt signaling pathway controls cell growth in many cell types by modulating the activity of FOXO transcription factors. But the role of FOXOs in regulating CD8 T cell memory remains unknown. We show that phosphorylation of Akt, FOXO and mTOR in CD8 T cells occurs in a dynamic fashion in vivo during an acute viral infection. To elucidate the potentially dynamic role for FOXO3 in regulating homeostasis of activated CD8 T cells in lymphoid and non-lymphoid organs, we infected global and T cell-specific FOXO3-deficient mice with Lymphocytic Choriomeningitis Virus (LCMV). We found that FOXO3 deficiency induced a marked increase in the expansion of effector CD8 T cells, preferentially in the spleen, by T cell-intrinsic mechanisms. Mechanistically, the enhanced accumulation of proliferating CD8 T cells in FOXO3-deficient mice was not attributed to an augmented rate of cell division, but instead was linked to a reduction in cellular apoptosis. These data suggested that FOXO3 might inhibit accumulation of growth factor-deprived proliferating CD8 T cells by reducing their viability. By virtue of greater accumulation of memory precursor effector cells during expansion, the numbers of memory CD8 T cells were strikingly increased in the spleens of both global and T cell-specific FOXO3-deficient mice. The augmented CD8 T cell memory was durable, and FOXO3 deficiency did not perturb any of the qualitative attributes of memory T cells. In summary, we have identified FOXO3 as a critical regulator of CD8 T cell memory, and therapeutic modulation of FOXO3 might enhance vaccine-induced protective immunity against intracellular pathogens.

## Author Summary

CD8 T cells are vital for controlling infections with viruses, intracellular bacteria and protozoa. Induction of T and B cell memory is the basis of vaccinations and cellular immunity to intracellular pathogens depends upon the number and quality of memory CD8 T cells. Understanding the mechanisms that control various facets of CD8 T cell memory is of fundamental importance for development of effective vaccines. In this study, we have identified the transcription factor FOXO3 as a crucial regulator of the magnitude of CD8 T cell memory. During a T cell response, FOXO3 limits the number of memory CD8 T cells by inhibiting the accumulation of memory precursor effector cells that give raise to long-lived CD8 T cells. Loss of FOXO3 activity in T cells led to a durable increase in the number of memory CD8 T cells, and the functional quality of FOXO3-deficient memory CD8 T cells was unaffected by FOXO3 deficiency. Thus, our studies suggest that targeting FOXO3 activity may be a fruitful strategy to augment vaccine-induced CD8 T cell memory and protective immunity.

## Introduction

The ability of the immune system to respond rapidly and vigorously to antigen re-exposure is termed immunological memory, which is one of the tenets of adaptive immunity [1], [2]. Induction of memory B and T cells is the basis of immunological memory induced by infections or vaccinations [2]–[4]. As compared to naïve T cells, memory T cells are hyper-reactive to antigenic stimulation and swiftly proliferate and/or differentiate into effector cells to confer protective immunity expeditiously [5]–[8]. The ability of memory T cells to confer protective immunity depends upon the number and quality of memory T cells [5], [9]–[13]. Understanding the mechanisms that regulate the quantity and quality of T cell memory is fundamentally important for the development of effective vaccines.

During a CD8 T cell response, engagement of the TCR, along with appropriate co-stimulatory and inflammatory signals, activate naïve T cells to proliferate and differentiate into effector cells [1], [4], [8], [13], [14]. In the case of LCMV infection, the peak of T cell expansion is reached at 8–10 days after infection, and the majority of the newly generated effector cells present at the peak of the response are short-lived and fated for deletion [15]–[17]. But, a small subset of the effectors, termed memory precursor effector cells (MPECs), possesses the potential to survive and differentiate into long-lived memory cells [16], [17]. The number of memory CD8 T cells generated depends largely upon the magnitude of the expansion of MPECs during the T cell response. Substantial progress has been made in deciphering the extracellular signals and transcription factors that regulate the differentiation of MPECs [1], but the signaling pathways that govern the number of MPECs, their differentiation into memory CD8 T cells, and the maintenance of CD8 T cell memory are not fully understood.

The FOXO family of transcription factors plays a crucial role in governing cellular proliferation, apoptosis, energy metabolism, and stress resistance in response to dynamic alterations in stress and abundance of nutrients and growth factors in many cell types [18]–[26]. In mammals, the FOXO family consists of at least four members: FOXO1, FOXO3, FOXO4, and FOXO6 [22], [27], [28]. The activity of FOXOs is regulated by post-translational modifications, most notably, phosphorylation [25], [26], [29]–[31]. In resting, quiescent cells, FOXOs exist in a hypo-phosphorylated state and localize to the nucleus to control the transcription of their target genes such as p27^Kip1^, p21^Cip1^, p300, BIM, Fas ligand that are involved in regulating cellular proliferation or apoptosis [25], [32]–[43]. However, in response to stimulation with growth factors or cytokines that stimulate the PI3K/AKT signaling pathway, activated Akt phosphorylates FOXO leading to its exclusion from the nucleus and degradation by proteolysis in the cytoplasm [18], [25], [29], [44], [45]. As a result, the transcription of FOXO target genes is diminished, which in turn facilitates cell cycle entry and/or survival. T cells express FOXO1 and FOXO3, and there has been a recent surge in interest to elucidate the importance of FOXOs in regulating T cell biology [46]–[53]. Phosphorylation-mediated inactivation of FOXOs and downregulation of p27^Kip1^ appear to be obligatory steps for T cells to enter the cell cycle in response to TCR engagement [24], [41], [53]. FOXO1 has been shown to control several aspects of T cells including the expression of adhesion molecules like L-selectin and CCR7, cytokine receptors like the IL-7 receptor, development of regulatory T cells, and protection against autoimmunity [51], [54]. Unlike FOXO1, the role of FOXO3 in T cell homeostasis is less well understood.

It was first reported that global deletion of FOXO3 results in lymphadenopathy and spontaneous activation of T cells, but other independent studies have failed to confirm these results, possibly due to differences in the genetic background of mutant mice [19], [40], [49]. Nonetheless, elegant studies from the Hedrick group have showed that FOXO3 inhibits the primary expansion of CD8 T cells in the spleen by regulating IL-6 production by dendritic cells [49]. However, the T cell intrinsic role of FOXO3 in regulating various phases of the polyclonal multi-epitope-specific CD8 T cell response to an acute viral infection in lymphoid and non-lymphoid organs remains to be determined. In this study, using global and conditional T cell-specific FOXO3 knockout mice, we have systematically examined the role of FOXO3 in regulating the: (1) expansion and function of polyclonal antigen-specific CD8 T cells in lymphoid and non-lymphoid organs; (2) antigen-driven in vivo proliferation of virus-specific CD8 T cells; (3) differentiation of CD8 T cells into short-lived effector cells (SLECs) and MPECs; (4) contraction of antigen-specific CD8 T cells in lymphoid and non-lymphoid organs; (5) the numbers and function of memory CD8 T cells; (6) proliferative renewal of memory CD8 T cells; (7) secondary CD8 T cell responses and protective immunity. These studies show that FOXO3 regulates the clonal expansion of polyclonal CD8 T cells in a tissue-specific fashion by T cell intrinsic mechanisms. The enhanced expansion of CD8 T cells was clearly not due to an increased proliferation rate, but was instead associated with reduced cellular apoptosis. Furthermore, we show that FOXO3 deficiency markedly enhances the size of the memory CD8 T cell compartment without affecting the phenotype or quality of memory CD8 T cells. These findings have implications in the design of effective vaccines that engender potent and effective protective cellular immunity against intracellular pathogens and tumors.

## Results

### Dynamic, in vivo phosphorylation of Akt, FOXO, and mTOR in antigen-specific CD8 T cells

**Figure 1:**
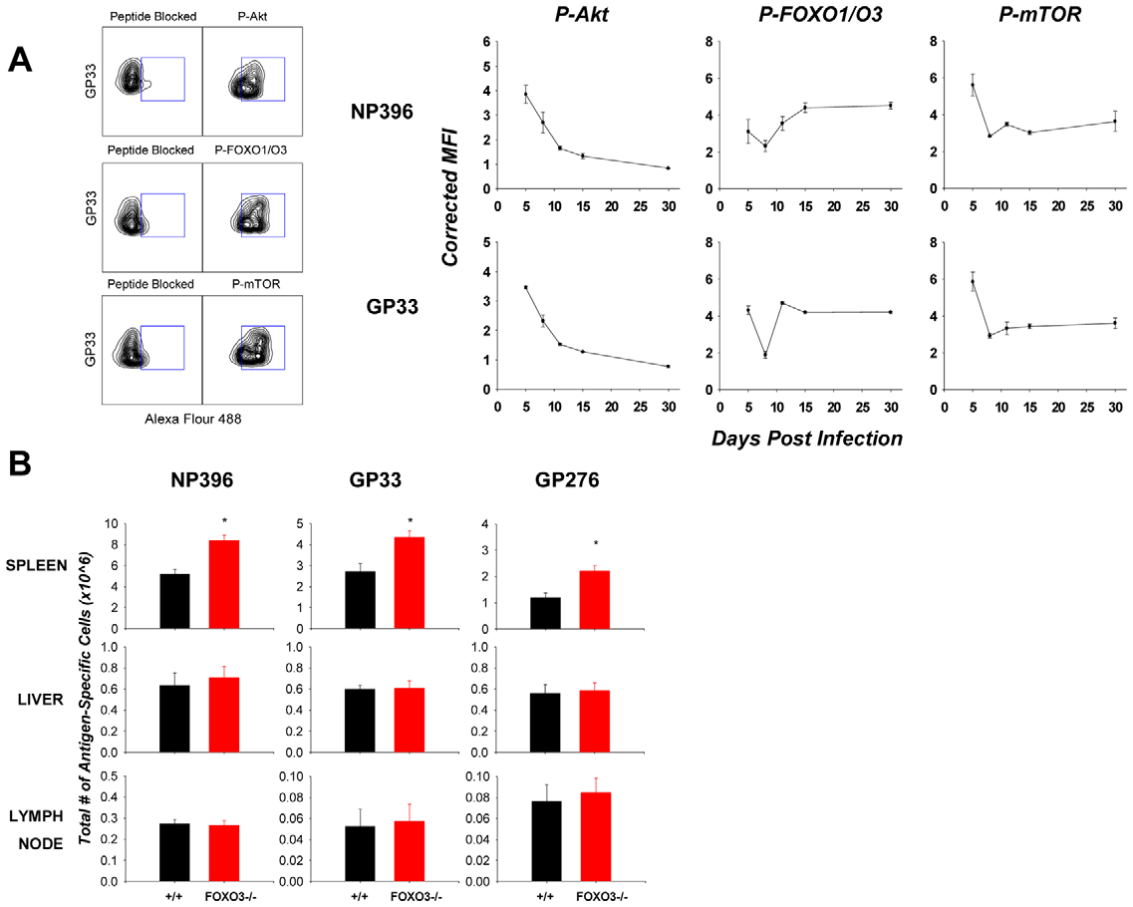
Loss of FOXO3 leads to a tissue-specific increase in expansion of effector CD8 T cells. (**A**) Dynamic, in vivo alterations in phosphorylation of FOXO- associated signaling proteins in LCMV-specific CD8 T cells. C57BL/6 mice were infected with LCMV, and at the indicated days PI, splenocytes were stained with anti-CD8, MHC-I tetramer (D^b^/NP396 or D^b^/GP33) and either the anti-P-Akt (T308), P-FOXO-1/3a (T24/T32), or P-mTOR (S2448) antibodies. As controls, these antibodies were pre-incubated with their specific antigenic peptide before adding on to the cells. Representative flow plots (left) gated on tetramer-binding CD8s from day 5 PI mice indicate specific antibody staining in relation to the peptide blocked controls. Plotted data (Corrected MFI) is expressed as the difference of observed MFI for the phospho-specific protein and peptide-blocked control (right), divided by the peptide-blocked control. Data are representative of at least three independent experiments. (**B**) +/+ and FOXO3−/− mice were infected with LCMV, and at 8 days PI, LCMV specific CD8 T cells were quantified in spleen, liver and lymph nodes by staining with anti-CD8 and MHC-I tetramers. Data are representative of 4 independent experiments with 4–6 mice/group/experiment.

FOXO3 has emerged as a key regulator in a number of physiological outcomes, including metabolism, ageing, and vascular reactivity [21]–[23], [27], [46], [55]. More recently, in the immune system, there is increasing evidence that FOXO3 is a critical regulator of T cell homeostasis [46], [47], [52], [53], [55]. While a number of signaling pathways and post translational modifications may be involved in controlling the activity of FOXO3, phosphorylation mediated through a PI3K-Akt centric signaling module primarily regulates FOXO3 function in T cells [29], [45], [56], [57]. In order to fully understand the role of the Akt/FOXO3 axis in the control of CD8 T cell homeostasis, we developed a phospho-specific, flow cytometric method to quantify in vivo phosphorylation levels of key proteins implicated in FOXO3 activity: the upstream kinase Akt, FOXO3 itself, as well as a potential downstream substrate of Akt, the kinase, mammalian target of Rapamycin (mTOR) (**Figure 1A**). **Figure 1A** illustrates the kinetics of phosphorylation of Akt (Thr308), FOXO1/3 (T24/T32), and mTOR (S2448) in antigen-specific CD8 T cells during an acute LCMV infection. The phosphorylation of Akt (Thr308) and mTOR (S2448) was highest at day 5 post-infection (PI), and subsided to steady-state levels by days 10 and 8 PI respectively. Interestingly, the phosphorylation dynamics for FOXO1/O3 were different from that of Akt and mTOR; the phosphorylation levels for FOXO1/O3 dropped between days 5 and 8 PI, but gradually increased back to steady-state levels by days 10–15 PI. Note that the phosphorylation kinetics of NP396-specific CD8 T cells after day 8 PI was slightly delayed, as compared to those in GP33-specific CD8 T cells. In summary, these findings demonstrate that the in vivo phosphorylation levels of Akt, FOXO1/O3, and mTOR are highly dynamic, and of note, the phosphorylation kinetics of Akt correlated with mTOR phosphorylation but not with phosphorylation of FOXO1/O3 during a T cell response to LCMV. However, the specific role of FOXO3 in regulating different phases of the CD8 T cell response has not been carefully examined.

### Regulation of CD8 T cell expansion by FOXO3 is tissue specific

Infection of mice with LCMV elicits a potent, multiple epitope-specific, CD8 T cell response wherein virus-specific CD8 T cells are distributed to both lymphoid and non-lymphoid organs [58], [59]. To examine the role of FOXO3 in regulating CD8 T cell responses to an acute viral infection, groups of wild type (+/+) and FOXO3-deficient (FOXO3−/−) mice were infected with LCMV. At day 8 PI, we quantified CD8 T cells that are specific to three immuno-dominant LCMV epitopes in lymphoid (spleen and lymph nodes) and non-lymphoid (liver) tissues. **Figure 1B** shows that the numbers of LCMV-specific CD8 T cells in spleens of FOXO3−/− mice were significantly (P<0.05) higher than in +/+ mice. Surprisingly, the numbers of virus-specific CD8 T cells in the lymph nodes and liver of FOXO3−/− mice were comparable to those in +/+ mice (**Figure 1B**). These data suggested that FOXO3 downregulates the accumulation of CD8 T cells in a tissue-specific fashion during an acute LCMV infection.

**Figure 2:**
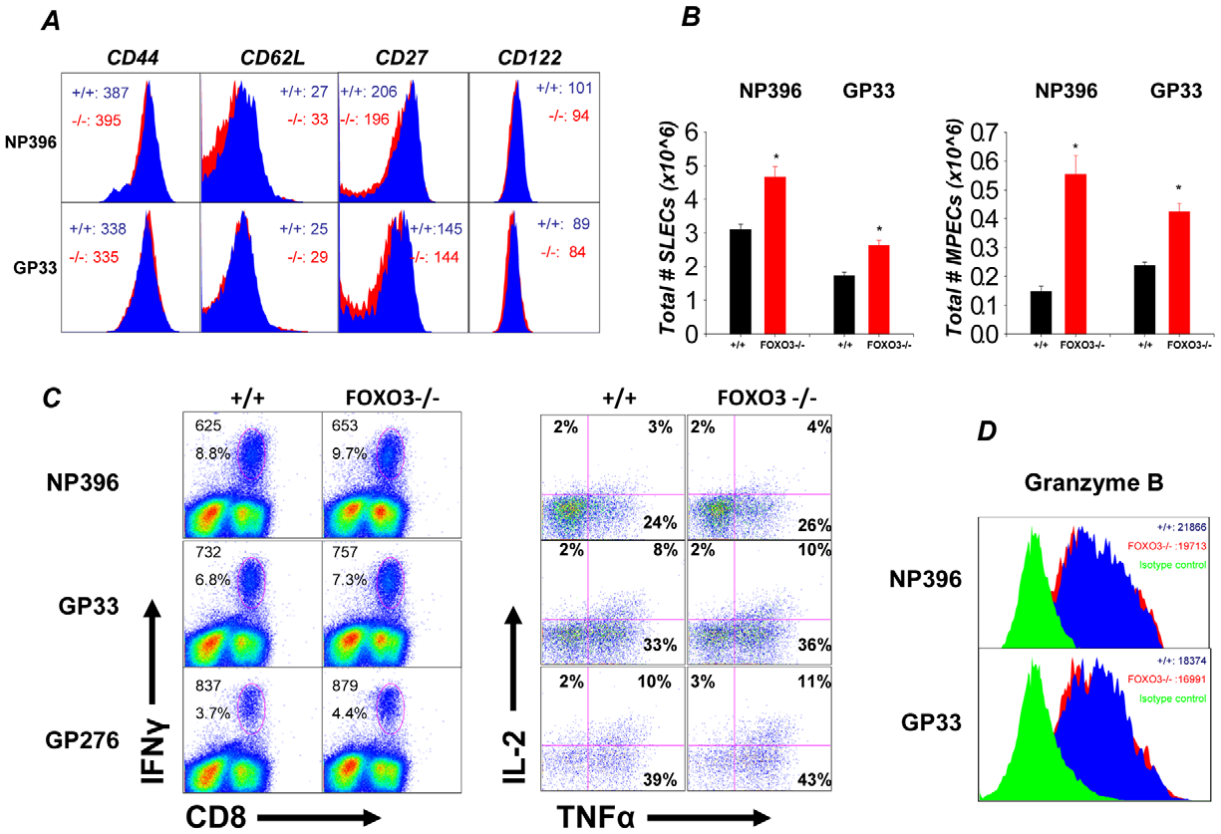
Absence of FOXO3 does not affect the phenotype or function of effector CD8 T cells. (**A**) At 8 days after LCMV infection, splenocytes from +/+ and FOXO3−/− mice were isolated and stained for expression of CD44, CD62L, CD27 and CD122 on NP396- (top) and GP33- (bottom) specific CD8 T cells. Representative histograms in panel A are gated on CD8 and MHC-I tetramer positive populations with the numbers indicating MFI for the indicated protein in either +/+ or FOXO3−/− mice. (**B**) Total splenocytes were stained with MHC-I tetramer, anti-CD8, anti-CD127 and anti-KLRG-1, and the total number of SLECs (KLRG-1^high^/CD127^low^) and MPECs (CD127^high^/KLRG-1^low^) were quantified by flow cytometry. Data are from 4 to 6 independent experiments with 3–6 mice/group/experiment; error bars represent the SEM and * indicates statistical significance at p<.05. (**C**) On day 8 PI, Splenocytes from +/+ or FOXO3−/− mice were stimulated with LCMV epitope peptides for 5 hours directly ex-vivo. Following stimulation, cells were stained for cell surface CD8 and intracellular IFNγ, IL-2 and TNFα. Panel **C** shows cytokine production by effector CD8 T cells. Representative dot plots (left) are gated on total lymphocytes with the top number indicating observed MFI for IFNγ staining in peptide-stimulated CD8 T cells and the bottom number indicating percentage of total splenocytes that are CD8 and IFNγ positive. Dot plots (right) represent the percentage of IL-2 and/or TNFα producing cells among IFNγ^+ve^ CD8 T cells. (**D**) Intracellular staining for Granzyme B. The FACS histograms are gated on LCMV-specific CD8 T cells from +/+ (BLUE) and FOXO3−/− (RED) mice. The green histogram represents staining with isotype control antibodies. The data are MFIs for Granzyme B expression +/− SD.

Next, we assessed whether FOXO3 deficiency affected the cell surface phenotype of LCMV-specific effector CD8 T cells at day 8 PI. The expression levels of CD44, CD62L, CD27, and CD122 on FOXO3−/− LCMV-specific CD8 T cells were comparable to those on +/+ CD8 T cells (**Figure 2A**). The population of LCMV-specific CD8 T cells in the spleen is comprised of at least two subsets of effector cells based on the cell surface expression of IL-7 receptor α (CD127) and KLRG-1: the SLECs (KLRG-1^HI^/ CD127^LO^) a majority of which are destined for apoptosis, and the MPECs (CD127^HI^/KLRG-1^LO^), which are poised to differentiate into memory CD8 T cells [16], [17], [60]. **Figure 2B** shows that FOXO3 deficiency significantly enhanced the absolute numbers of both SLECs and MPECs in the spleen at day 8 PI. Of note is the marked increase in the total number of MPECs in FOXO3−/− mice. In summary, these data suggested that FOXO3 deficiency increased the clonal expansion of CD8 T cells without disrupting the differentiation of SLECs and MPECs.

### FOXO3 deficiency does not affect the function of effector CD8 T cells

To detect possible differences in the functionality of antigen-specific CD8 T cells from +/+ and FOXO3−/− mice, we assessed their ability to produce the cytokines IFNγ, TNFα, and IL-2 in response to antigenic stimulation directly ex vivo (**Figure 2C**). The MFIs of IFNγ staining for FOXO3−/− and +/+ effector CD8 T cells were similar (**Figure 2C**). Additionally, the percentages of epitope-specific, CD8 T cells that produced two cytokines (IFNγ and TNFα) or three cytokines (IFNγ, TNFα, and IL-2) in FOXO3−/− mice were similar to those in +/+ mice (**Figure 2C**). The MFIs for TNFα and IL-2 were also comparable between +/+ and FOXO3−/− CD8 T cells (data not shown). As a surrogate marker of the lytic function of effector CD8 T cells, we compared granzyme B expression between +/+ and FOXO3−/− LCMV-specific effector CD8 T cells directly ex vivo. The levels of granzyme B in FOXO3−/− CD8 T cells were similar to those in +/+ CD8 T cells (**Figure 2D**). Taken together, data in **Figures 1** and **2** suggested that FOXO3 deficiency increased the expansion of virus-specific CD8 T cells without affecting their phenotype or function. Consistent with normal CD8 T cell effector function, LCMV control in FOXO3−/− mice was similar to that in +/+ mice (not shown).

### Increased accumulation of CD8 T cells in FOXO3−/− mice is linked to reduced apoptosis and not enhanced proliferation

**Figure 3:**
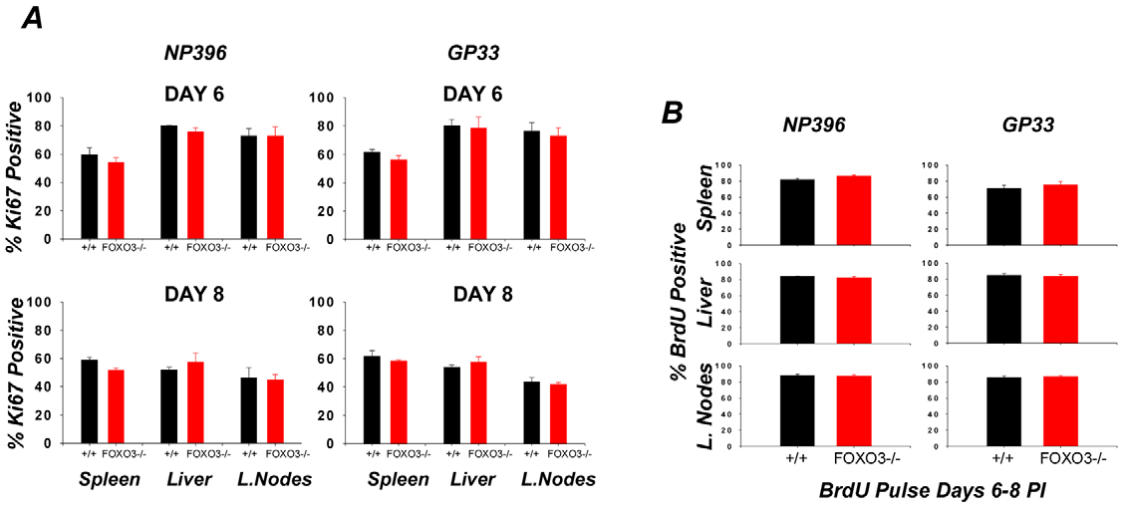
In vivo proliferation of LCMV-specific CD8 T cells. (**A**) At either 6 (top) or 8 (bottom) days PI, lymphocytes from spleen, liver and lymph nodes were collected and stained with anti-CD8, MHC-I tetramers and anti-Ki67. The percentage of Ki67 positive cells amongst tetramer positive CD8 T cells was determined by flow cytometry. Data are the mean of at least 9 mice from 2 experiments. (**B**) +/+ and FOXO3−/− mice were administered BrdU between days 6–8 PI. At day 8 PI, lymphocytes from spleen, liver and lymph nodes were collected and stained with anti-CD8, MHC-I tetramers and anti-BrdU. The percentage of BrdU -positive cells amongst tetramer positive CD8 T cells was determined by flow cytometry. Data are the mean of 6 mice per group from 2 independent experiments.

To rigorously address whether the greater number of LCMV-specific CD8 T cells in the spleen of FOXO3−/− mice was due to increased proliferation, we utilized two different approaches, Ki67 staining and BrdU incorporation. First, we quantified proliferation of LCMV-specific CD8 T cells by staining for the nuclear antigen Ki67 directly ex vivo (**Figure 3A**) during the peak clonal expansion phase (days 6 and 8 PI) of the CD8 T cell response. For the second approach, we measured BrdU incorporation by LCMV-specific CD8 T cells in vivo from days 6–8 PI (**Figure 3B**). As shown in **Figures 3A and 3B**, the percentages of of Ki67^+ve^ LCMV-specific CD8 T cells and BrdU^+ve^ LCMV epitope-specific CD8 T cells in spleens, liver, and lymph nodes of FOXO3−/− mice were similar to those in +/+ mice. Measurement of Ki67 expression at day 5 PI also showed that FOXO3 deficiency did not alter the proliferation of LCMV-specific CD8 T cells (**Figure S1**) Taken together, these data indicated that enhanced proliferation was not sufficient to explain the increase in antigen-specific CD8 T cells in FOXO3−/− mice during LCMV infection.

**Figure 4:**
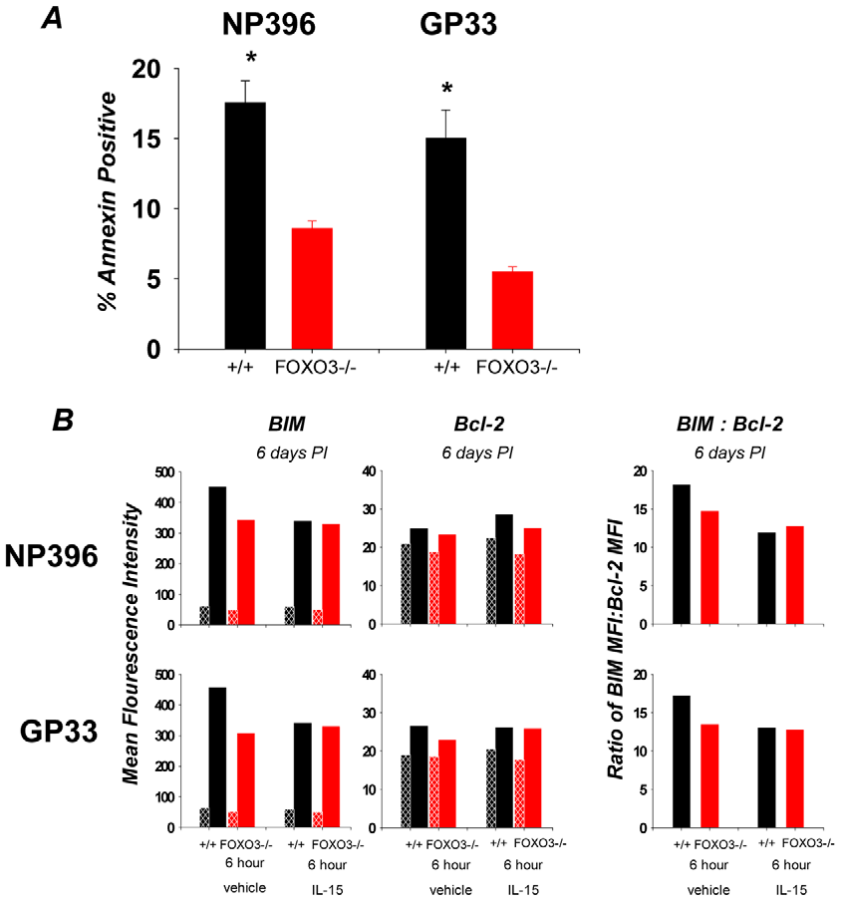
Activated CD8 T cells in FOXO3−/− mice display decreased apoptosis. To address whether the increase in antigen-specific cells observed in FOXO3−/− mice at day 8 was in fact the result of decreased apoptosis, we assessed the relative expression levels of a number of cell survival or cell death markers. (**A**) Splenocytes isolated from day 6 PI mice were stained with anti-CD8, MHC-I tetramers and Annexin V directly ex vivo. Data is expressed as percentage of Annexin V positive cells amongst tetramer-binding CD8 T cells. Data are from at least two independent experiments with 4 mice/group/experiment; error bars represent the SEM and * indicates statistical significance at p<.005 (**B**) At day 6 PI, splenocytes from +/+ and FOXO3−/− mice were cultured for 6 hours with or without IL-15. After culture, cells were stained with MHC I tetramers, anti-CD8, anti-BIM, and anti-Bcl-2. The MFI of staining for BIM and Bcl-2 in tetramer-binding CD8 T cells was assessed by flow cytometry (left and middle); adjacent checkered bars are the MFIs with isotype control antibodies. In **B**, bar graphs on the right show the ratios of BIM:Bcl-2 MFI.

CD8 T cell homeostasis is not simply determined by alterations in proliferation, it is also regulated by alterations in cell death. During the clonal expansion phase of the CD8 T cell response, there is concomitant proliferation and apoptosis [61], and therefore the magnitude of clonal expansion is dependent upon the relative rates of proliferation and apoptosis. To assess cellular apoptosis, we determined the percentages of Annexin V^HI^ LCMV-specific CD8 T cells in spleens of +/+ and FOXO3−/− mice at day 6 PI, directly ex vivo. Data in **Figure 4A** shows that the percentages of Annexin V^HI^ LCMV-specific CD8 T cells in spleens of FOXO3−/− mice were significantly lower than in spleens of +/+ mice. These data suggested that FOXO3 controls the accumulation of effector CD8 T cells by promoting cellular apoptosis during an acute LCMV infection. During antigen-driven proliferation, the competing pro-apoptotic effects of TGF-β and the anti-apoptotic effects of IL-15 regulate the apoptotic rate of CD8 T cells [61]. Because apoptosis of LCMV-specific CD8 T cells was dampened by FOXO3 deficiency (**Figure 4A**), and IL-15 has been reported to induce phosphorylation of FOXO3 [62], we hypothesized that FOXO3 might downregulate clonal expansion by reducing the viability of CD8 T cells that are deprived of IL-15. The balance in the levels of the anti-apoptotic molecule Bcl-2 and the pro-apoptotic molecule BIM controls the susceptibility of a T cell to apoptotic stimuli [36], [38], [39], [63]–[65]. Since BIM is a target gene for FOXO3, we tested whether deficiency of FOXO3 might lead to lower expression of BIM in proliferating CD8 T cells (day 6 PI), by comparing the levels of BIM in +/+ and FOXO3−/− LCMV-specific CD8 T cells after culture with or without IL-15. As shown in **Figure 4B** and **Figure S2**, BIM expression in +/+ CD8 T cells was higher than in FOXO3−/− CD8 T cells, when cultured in media without IL-15. However, IL-15 reduced BIM expression in +/+ CD8 T cells to levels seen in FOXO3−/− CD8 T cells; IL-15 did not affect BIM expression in FOXO3−/− CD8 T cells. These data suggested that FOXO3 might control BIM expression in IL-15-deprived CD8 T cells. When analyzing for BIM in direct relation to Bcl-2, we observed that after 6 days of infection, LCMV-specific CD8 T cells from +/+ mice exhibit an increased BIM to Bcl-2 ratio, as compared to FOXO3−/− CD8 T cells (**Figure 4B**). Thus, we propose that FOXO3 might downregulate the accumulation of proliferating CD8 T cells by inducing BIM expression.

### Contraction of CD8 T cells in FOXO3−/− mice

**Figure 5:**
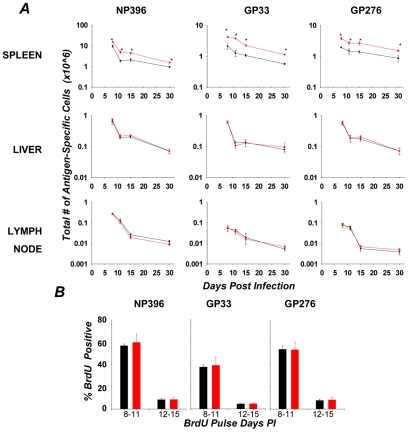
Effect of FOXO3 deficiency on contraction of CD8 T cells. (**A**) +/+ and FOXO3−/− mice were infected with LCMV and at the indicated days PI, cells from spleen, liver and lymph nodes were stained with anti-CD8 and MHC-I tetramers. Data are representative of 3 to 8 independent experiments with 4–6 mice/group/experiment for each indicated time point. (**B**) +/+ and FOXO3−/− mice were infected with LCMV and given a BrdU injection at either 8 or 12 days PI and administered BrdU in drinking water between either days 8–11 or 12–15 PI. At the end of each BrdU pulse (day 12 or day 15), splenocytes were stained with anti-CD8, MHC-I tetramers and anti-BrdU. The percentage of BrdU positive cells amongst tetramer binding CD8 T cells for each pulse (8–11, or 12–15 days PI) was determined by flow cytometry. Data are the mean of at least 6 +/+ or FOXO3−/− mice/group.

Next, we examined whether FOXO3 deficiency regulated the contraction of CD8 T cells in lymphoid and non-lymphoid tissues during an acute LCMV infection. Virus-specific CD8 T cells were quantified in spleen, liver, and lymph nodes at days 8, 11, 15, and 30 PI (**Figure 5A**). Overall, the slopes of the contraction curves for LCMV-specific CD8 T cells in FOXO3−/− mice were comparable to those of +/+ mice. Thus, the contraction of LCMV-specific CD8 T cells was minimally affected by FOXO3 deficiency. Next, we assessed whether FOXO3 regulated proliferation of LCMV-specific CD8 T cells during the contraction phase. In vivo BrdU incorporation studies showed that the percentages of LCMV-specific CD8 T cells that incorporated BrdU between days 8–11 or 12–15 PI in FOXO3−/− mice were similar to those in +/+ mice (**Figure 5B**).

### FOXO3 deficiency enlarges the size of the memory CD8 T cell compartment in a tissue-specific fashion

**Figure 6:**
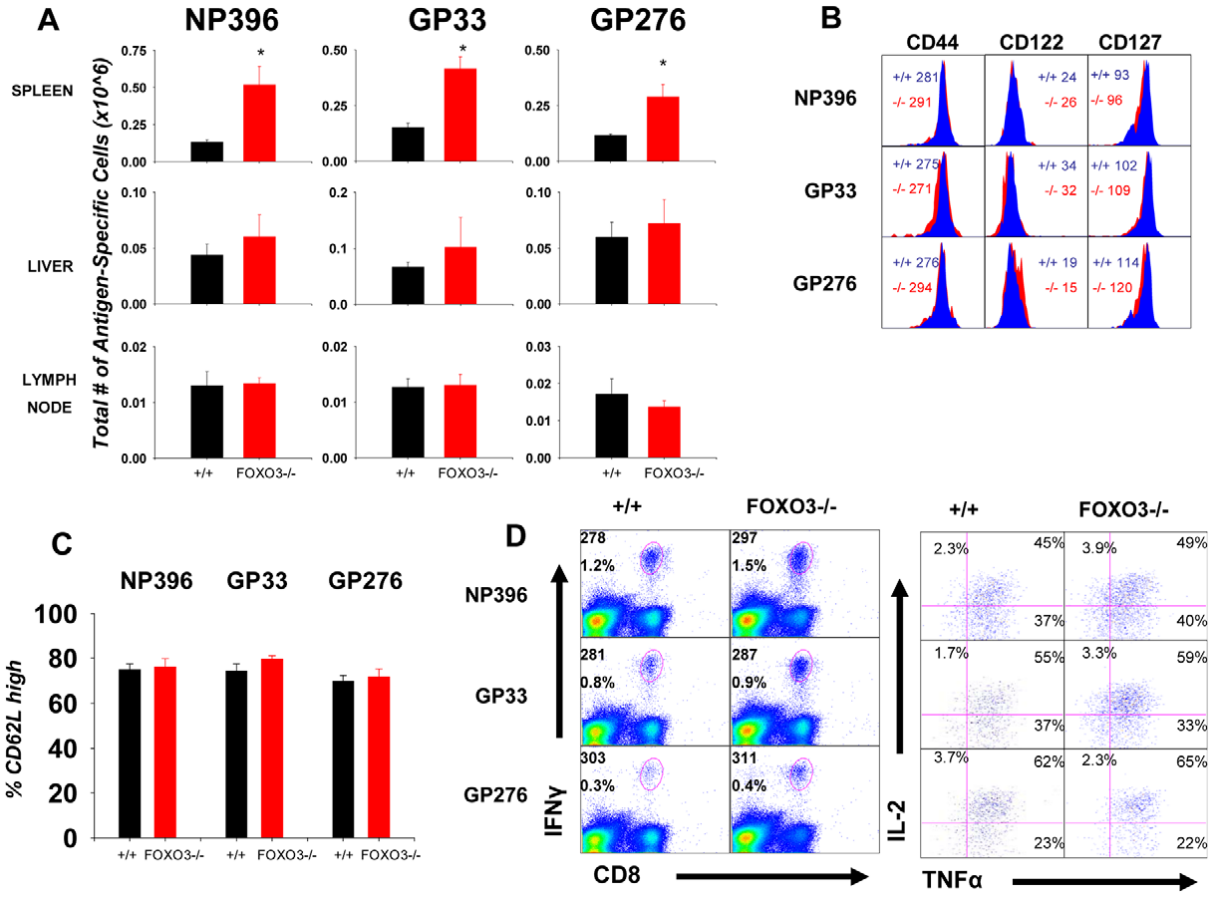
FOXO3 deficiency leads to a *tissue-specific* increase in the size of the CD8 T cell memory compartment with no effect on the quality of memory CD8 T cells. (**A**) +/+ and FOXO3−/− mice were infected with LCMV, and virus-specific CD8 T cells were quantified by flow cytometry at 180 days PI by staining with anti-CD8 and MHC-I tetramers. Data are from two independent experiments with 5–7 mice/group/experiment; error bars represent the SEM and * indicates statistical significance at p<.03. (**B**) At 180 days PI, LCMV-specific CD8 T cells in +/+ and FOXO3−/− mice were examined for expression of CD44, CD122 and CD127. Representative plots are gated on tetramer-binding CD8 T cells. The numbers represent MFI of staining for each marker in +/+ or FOXO3−/− mice. (**C**) Splenocytes were stained with MHC-I tetramer, anti-CD8, and anti-CD62L. The percentage of LCMV-specific CD8 T cells with high expression of CD62L was determined by flow cytometry and graphed accordingly. Error bars represent the S.E.M from at least 3 independent experiments with 4 mice/group/experiment. (**D**) Cytokine production by LCMV-specific memory CD8 T cells was assessed by intracellular cytokine staining. Representative dot plots are gated on total splenocytes, with the top number indicating the MFI for IFNγ and the bottom number denoting the percentage of total splenocytes that are CD8 and IFNγ positive (left). The percentage of double positive (IFNγ^+ve^ and IL-2^+ve^ or IFNγ^+ve^ and TNFα^+ve^) and triple positive (IFNγ^+ve^, IL-2^+ve^, and TNFα^+ve^) populations (right) is shown for the indicated epitope.

The number of memory CD8 T cells is a function of the magnitude of expansion and contraction during the T cell response [5]. Here, we determined whether increased clonal expansion of MPECs in FOXO3−/− mice (**Figure 2B**) translated to inflation of LCMV-specific memory CD8 T cells in lymphoid and non-lymphoid organs. We observed that FOXO3−/− mice exhibit a substantial increase in the numbers of NP396- (P<0.001), GP33- (P<0.01), and GP276- (P<0.04) specific CD8 T cells in spleen at 180 days PI (**Figure 6A**). Interestingly, there was no detectable increase in the numbers of LCMV-specific memory CD8 T cells in either the liver or lymph nodes at day 180 PI (**Figure 6A**). It should be noted that the magnitude of increase in the number of memory CD8 T cells in FOXO3−/− mice reflected the increased accumulation of MPECs during the primary response (**Figure 2B**). High numbers of memory CD8 T cells were maintained in FOXO3−/− mice stably until at least day 300 PI (data not shown). These data strongly imply that FOXO3 plays an important role in downregulating the magnitude of CD8 T cell memory in the spleen following an acute viral infection.

Phenotypic analysis of LCMV-specific memory CD8 T cells in FOXO3−/− and +/+ mice suggested that FOXO3 deficiency did not affect the expression of molecules that control T cell trafficking (CD44) or cytokine receptors (CD122 and CD127)(**Figure 6B**). In addition, assessment of CD62L levels on antigen specific CD8 T cells indicated that both central (CD62L^high^) and effector (CD62L^low^) memory frequencies were unaffected by FOXO3 deficiency (**Figure 6C**). Furthermore, functional analysis of antigen-triggered cytokine production did not reveal alterations in cytokine producing ability of LCMV-specific memory CD8 T cells from FOXO3−/− mice when compared to their +/+ counterparts (**Figure 6D**). In summary, data presented in **Figure 6** showed that FOXO3 deficiency increased the quantity of CD8 T cell memory without affecting the quality.

**Figure 7:**
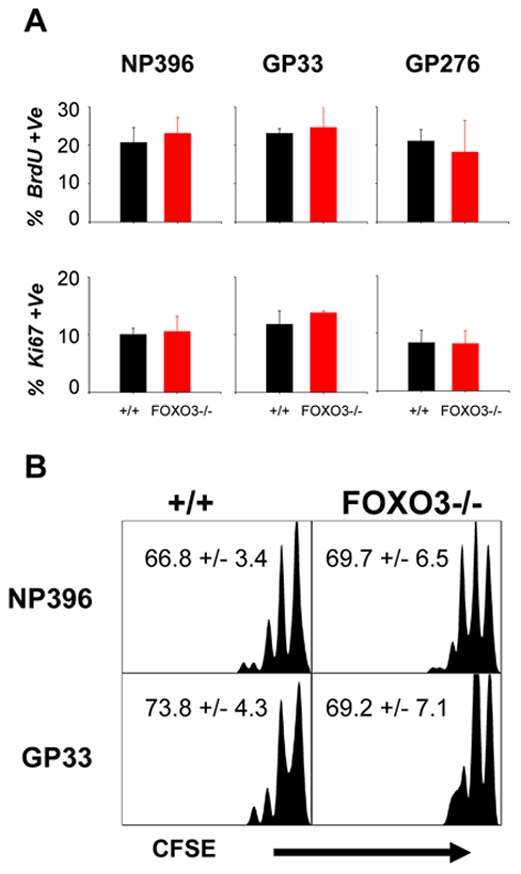
FOXO3 deficiency does not alter the homeostatic turnover of antigen- specific memory CD8 T cells. (**A**) +/+ and FOXO3−/− mice were infected with LCMV and given a BrdU injection at 282 days PI and administered BrdU in drinking water for the next 8 days. At the end of an 8 day BrdU pulse, splenocytes were stained with anti-CD8, MHC-I tetramers and anti-BrdU or anti-Ki67. The percentages of BrdU (top) or Ki67 (bottom) -positive cells amongst tetramer positive CD8 T cells were determined by flow cytometry. Graphs represent data from at least 4 mice/group. (**B**) To further investigate whether FOXO3 plays a role in homeostatic turnover, at 230 days PI, T cells were purified from the spleens of +/+ and FOXO3−/− mice, labeled with CFSE and adoptively transferred into naïve congenic B6/Ly5.1 mice. Thirty days after transfer, the dilution of CFSE in donor LCMV-specific, CD8 T cells was measured by flow cytometry. Representative histograms are gated on Ly5.2^+ve^ CD8 T cells that are specific for either the NP396 (top) or GP33 (bottom) epitope. Numbers represent percentages of divided cells.

It is well established that memory CD8 T cells are maintained for extended periods of time by proliferative renewal, driven by homeostatic cytokines IL-7 and IL-15 [11], [56], [66]–[68]. Although it is known that IL-7 and IL-15 signaling triggers phosphorylation of FOXO3 [56], [62], the effect of FOXO3 deficiency on proliferative renewal of memory CD8 T cells has not been examined. Using three approaches, we compared the cytokine-driven proliferative renewal of memory CD8 T cells in +/+ and FOXO3−/− mice. First, in vivo BrdU incorporation studies showed that the percentages of BrdU^+ve^ LCMV-specific memory CD8 T cells in FOXO3−/− mice were comparable to those in +/+ mice (**Figure 7A**). Likewise, the percentages of Ki67^+ve^ LCMV-specific CD8 T cells were unaffected by FOXO3 deficiency (**Figure 7A**). To further examine the effect of FOXO3 deficiency on the proliferative renewal of memory CD8 T cells, CD8 T cells from the spleens of LCMV-immune +/+ and FOXO3−/− mice were labeled with CFSE and adoptively transferred into congenic uninfected mice. Thirty days after cell transfer, flow cytometric analysis of CFSE staining revealed that donor LCMV-specific CD8 T cells from +/+ and FOXO3−/− mice proliferated equally in the recipient mice (**Figure 7B**). Taken together, data in **Figure 7** provided strong evidence that FOXO3 deficiency did not alter the homeostatic turnover of LCMV-specific memory CD8 T cells.

### Secondary CD8 T cell responses and protective immunity in FOXO3−/− mice

**Figure 8:**
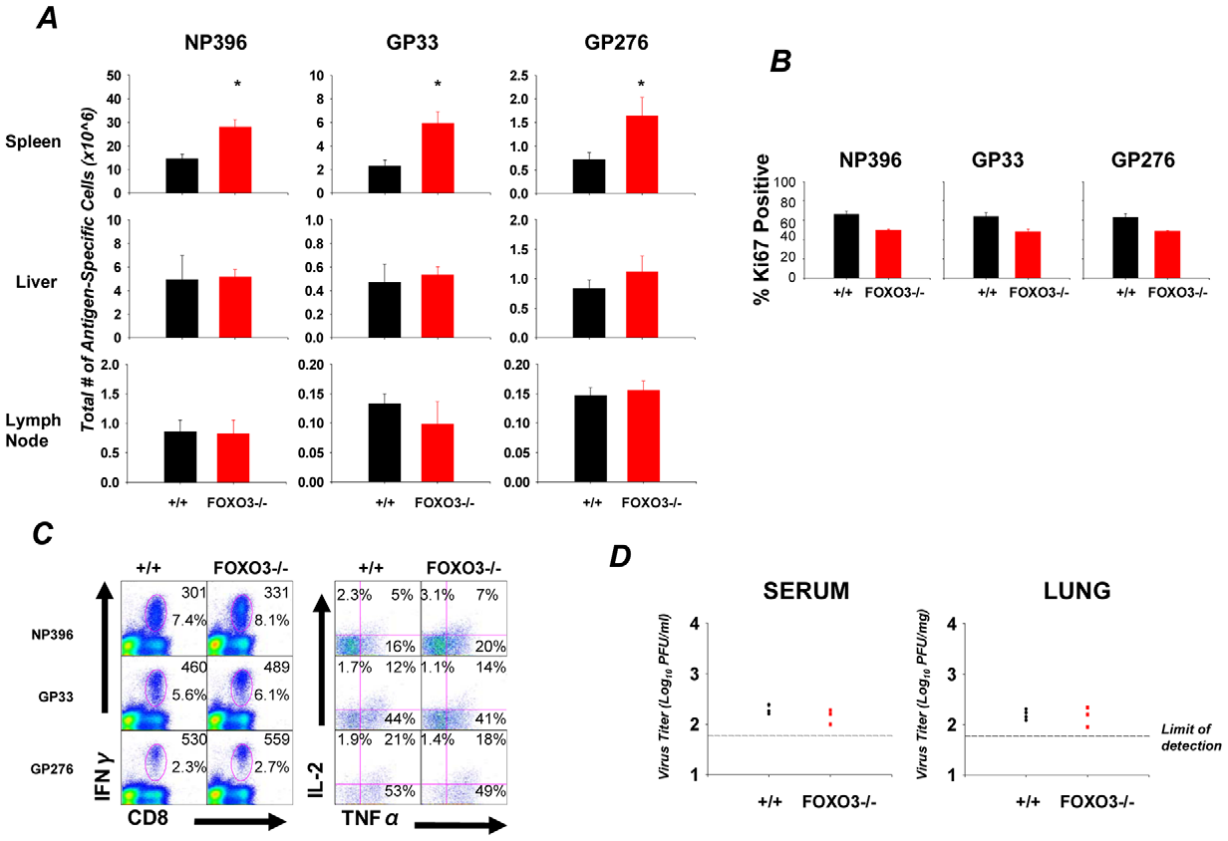
The secondary CD8 T cell response in FOXO3 deficient mice. +/+ and FOXO3−/− mice were infected with LCMV-Armstrong and after 90 days PI, these mice were challenged with 2.5×10^6^ PFU of LCMV clone 13. (**A**) Five days after LCMV clone13 infection, mononuclear cells from spleen, liver and lymph nodes were collected and the total number of LCMV-specific CD8 T cells was determined by staining with anti-CD8 and LCMV-specific MHC-I tetramers. Data are from 4–6 mice/group; error bars represent the SEM and * indicates statistical significance at p<.05. (**B**) Splenocytes from +/+ or FOXO3−/− mice were stained with anti-CD8, MHC-I tetramers and anti-Ki67. The percentage of Ki67 positive cells amongst tetramer positive CD8 T cells was determined by flow cytometry. (**C**) Splenocytes were stimulated ex vivo with LCMV-specific peptides for 5 hours. Following stimulation, cells were stained for surface expression of CD8 and intracellular expression of IFNγ, IL-2 and TNFα. The top number in the plots on the left is the MFI of staining for IFNγ. The bottom number in the plots indicates the percentage of total splenocytes that are CD8 and IFNγ positive. The plots on the right are gated on IFNγ^+ve^ CD8 T cells and the numbers are the percentages of cells that produced IFNγ and IL-2, IFNγ and TNFα, or IFNγ, IL-2, and TNFα. (**D**) LCMV titers in serum and lung were determined by plaque assay using a vero cell monolayer. Each symbol represents the viral titer of an individual mouse.

To observe the effect of FOXO3 deletion on recall responses of memory CD8 T cells and protective immunity, LCMV-immune +/+ and FOXO3−/− mice were challenged with LCMV clone 13, a strain of LCMV that establishes a chronic infection in naïve immunocompetent mice. At day 5 after challenge, the numbers of NP396- (P<0.01), GP33- (P<0.01) and GP276- (P<0.03) specific CD8 T cells in FOXO3−/− mice (**Figure 8A**) were significantly higher than in +/+ mice. The increased number of LCMV-specific CD8 T cells in spleens of LCMV clone 13-Challenged FOXO3−/− mice correlated with the increased numbers of memory CD8 T cells (**Figure 6A**). As in a primary infection (**Figure 1B**), we did not see a statistically significant increase in the numbers of LCMV-specific CD8 T cells in either the liver or lymph nodes (**Figure 8A**). Staining for Ki67 illustrated that FOXO3 deficiency did not affect the proliferation of LCMV-specific CD8 T cells during a secondary response (**Figure 8B**). Secondary effector CD8 T cells in spleens of FOXO3−/− mice produced comparable levels of IFNγ, TNFα, and IL-2 (**Figure 8C**). LCMV titers in tissues were comparable in +/+ and FOXO3−/− mice, which indicated that protective immunity was not compromised in the absence of FOXO3 (**Figure 8D**).

### FOXO3 regulates CD8 T cell Expansion by T cell intrinsic mechanisms

**Figure 9:**
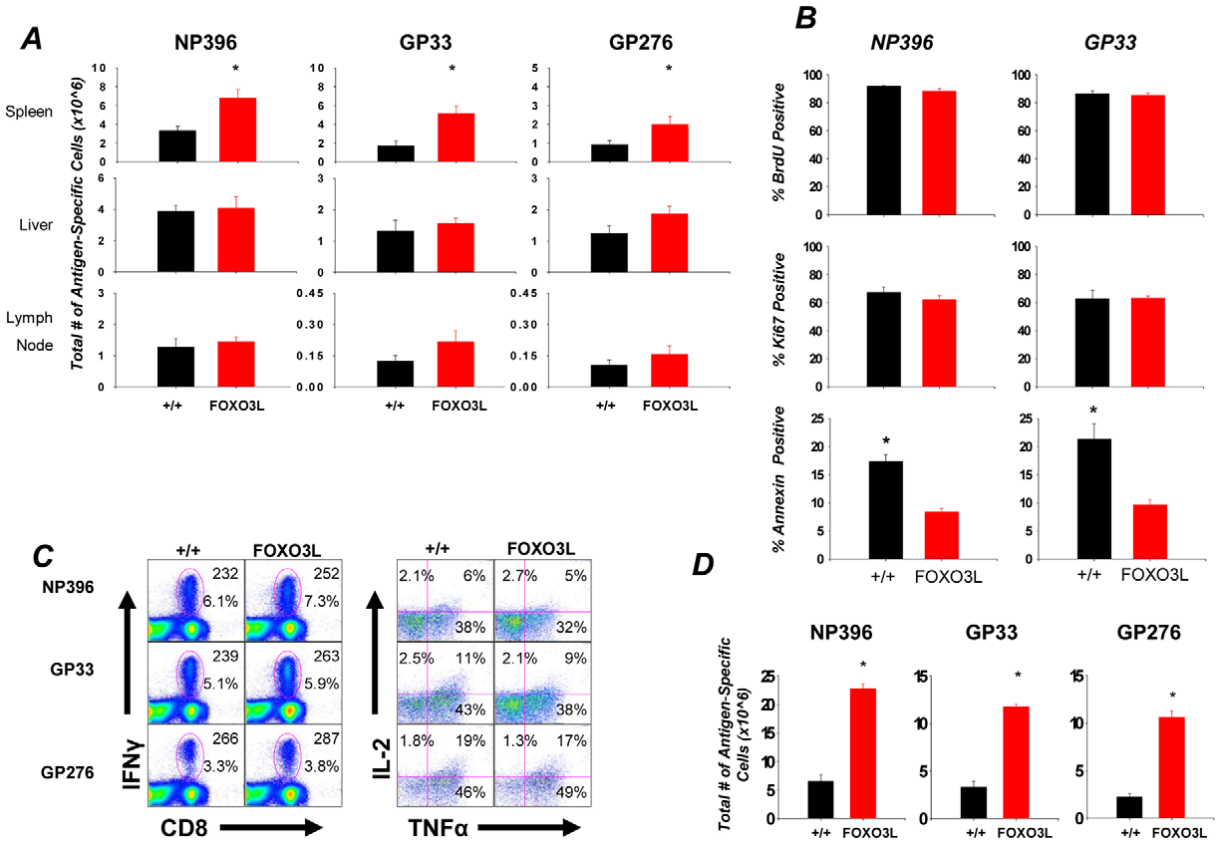
FOXO3 regulates CD8 T cell expansion through T cell-intrinsic mechanisms. (**A**) +/+ and FOXO3L mice were infected with LCMV. At 8 days PI, mononuclear cells from spleen, liver and lymph nodes were stained with anti-CD8 and MHC-I tetramers. Data are from 3–4 independent experiments with 4–6 mice/group/experiment; error bars represent the SEM and * indicates statistical significance at p<.03. (**B**) Investigation of in vivo proliferation and apoptosis by BrdU incorporation/Ki67 staining and Annexin V staining. +/+ and FOXO3L mice were infected with LCMV and given a BrdU injection at 6 days PI and administered BrdU in drinking water between days 6–8 PI. At the end of the BrdU pulse, splenocytes were stained with anti-CD8, MHC-I tetramers and anti-BrdU. The percentage of BrdU positive cells amongst tetramer positive CD8 T cells was determined by flow cytometry (top panel). In parallel studies, mononuclear cells were stained with anti-CD8, MHC-I tetramer and anti-Ki67 (middle panel) or Annexin V (lower panel). The percentage of Ki67 or Annexin V positive cells amongst tetramer positive CD8 T cells was determined by flow cytometry. Data are from 2–3 independent experiments; Graphs represent data from 6–8 mice/group/experiment; error bars represent the SEM and * indicates statistical significance at p<.001. (**C**) At day 8 PI, splenocytes were stimulated in vitro with LCMV specific peptides for 5 hours. Following stimulation, cells were stained for surface expression of CD8 and intracellular expression of IFNγ, IL-2 and TNFα. Representative dot plots (left) are gated from total lymphocytes with the top numbers indicating the MFI for IFNγ on CD8 T cells while the bottom number in the plot indicates the percentage of total splenocytes that are CD8 and IFNγ positive. Dot plots (right) represent the percentages of IL-2 and/or TNFα producing cells among IFNγ^+ve^ CD8 T cells. Representative plots from 1 of 6 individual experiments are illustrated. (**D**) To deplete CD4 T cells, +/+ and FOXO3L mice were injected with 100 ug of the monoclonal antibody, GK1.5, at days 0 and 4 relative to LCMV infection. At day 8 PI, splenocytes were stained with anti-CD8 and MHC-I tetramers to determine the total number of LCMV-specific CD8 T cells. Bars represent data collected from at least 4 mice; error bars represent the SEM and * indicates statistical significance at p<.01.

To address whether FOXO3 has a T cell intrinsic role in regulating polyclonal CD8 T cell responses to LCMV, we used a cre-loxP knockout strategy to generate the FOXO3L mice that lacked FOXO3 only in T cells [69], [70]. T cell-specific loss of FOXO3 in FOXO3L mice was confirmed by western blot and flow cytometry (**Figure S3**). FOXO3L and littermate +/+ mice were infected with LCMV and virus-specific CD8 T cell responses were analyzed at day 8 PI. In the spleen, FOXO3L mice exhibited a statistically significant increase in the numbers of LCMV-specific CD8 T cells (NP396 P<0.02; GP33 P<0.01; GP276 P<0.04) over their +/+ littermate controls (**Figure 9A**). It should be noted that the observed increase in the expansion of CD8 T cells in global FOXO3−/− mice (**Figure 1B**) was fully recapitulated in FOXO3L mice (**Figure 9A**). To assess whether greater accumulation of effector CD8 T cells in spleens of FOXO3L mice was driven by increased proliferation, we measured Ki67 expression and BrdU incorporation during the clonal expansion phase of the CD8 T cell response to LCMV. At day 6 PI, the percentages of both Ki67^+ve^ and BrdU^+ve^ LCMV-specific CD8 T cells in FOXO3L mice were comparable to those in +/+ mice, which suggested that enhanced accumulation of effector CD8 T cells in FOXO3L mice are not linked to an altered proliferation rate (**Figure 9B**). Likewise, percentages of Ki67^+ve^ CD8 T cells at day 5 PI were comparable in +/+ and FOXO3L mice (**Figure S4**). However, the percentages of Annexin V^HI^ LCMV-specific CD8 T cells in spleens of FOXO3L mice were significantly lower than in +/+ mice (**Figure 9B**). These data suggested that FOXO3 controls the accumulation of CD8 T cells during a primary response by regulating apoptosis of proliferating cells. Conditional deficiency of FOXO3 in T cells did not affect the cell surface phenotype (data not shown) or antigen-triggered cytokine production by LCMV-specific effector CD8 T cells at day 8 PI (**Figure 9C**).

Since both CD8 and CD4 T cells lack FOXO3 activity in FOXO3L mice, it could be argued that increased clonal expansion of CD8 T cells might result from enhanced CD4 T cell help in FOXO3L−/− mice. To address this issue, we depleted CD4 T cells in +/+ and FOXO3L mice and quantified CD8 T cell responses to LCMV in the absence of CD4 T cells. At day 8 PI, >95% of the CD4 T cells were depleted in spleen of both +/+ and FOXO3L mice (data not shown). The number of LCMV-specific CD8 T cells in spleen of CD4-depleted FOXO3L mice was substantially higher than in CD4 T cell-depleted +/+ mice (**Figure 9D**). Thus, in the apparent absence of CD4 T cells, FOXO3 deficiency in CD8 T cells was sufficient to increase the accumulation of virus-specific CD8 T cells during an acute LCMV infection.

### T cell-specific deletion of FOXO3 enhances the magnitude of CD8 T cell memory

**Figure 10:**
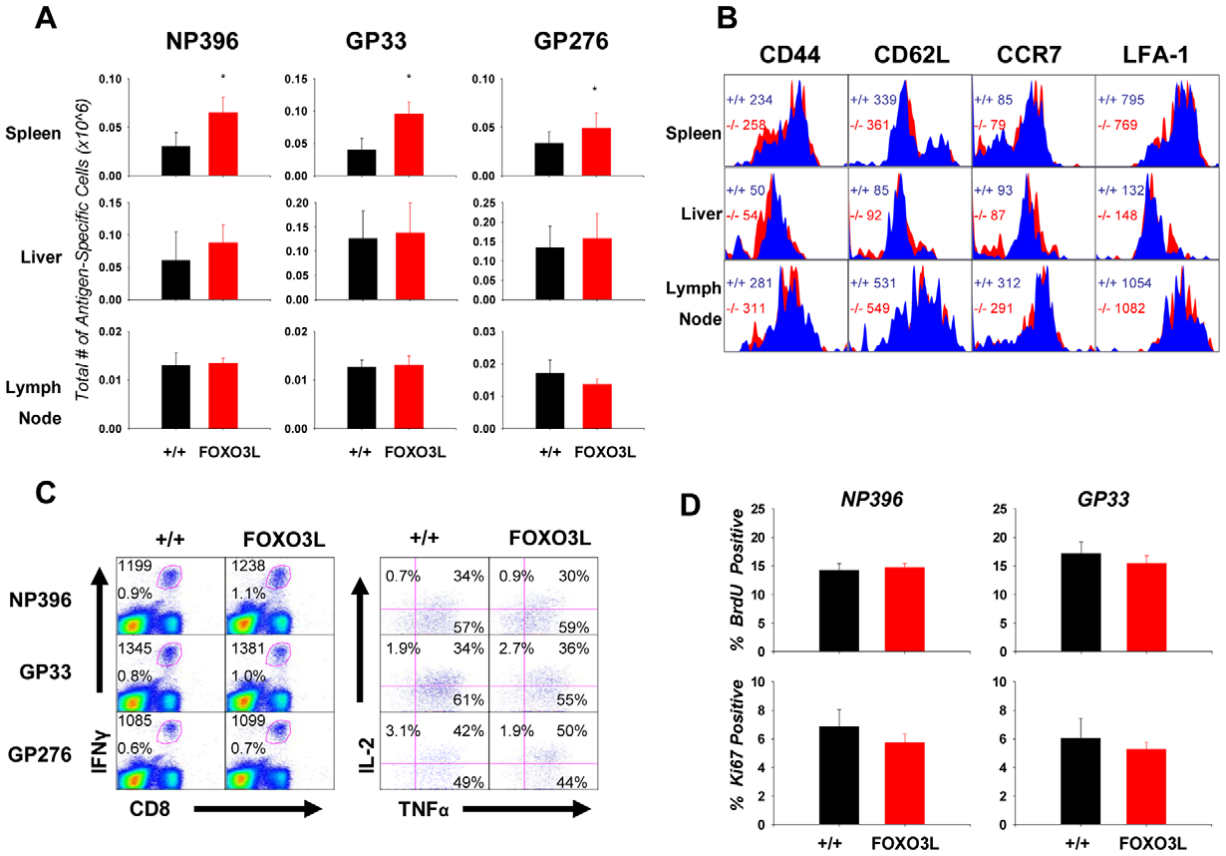
Loss of FOXO3 in the T cell compartment enhances the quantity of memory CD8 T cells without decreasing their quality. (**A**) At 180 days after LCMV infection, mononuclear cells from spleen, liver and lymph nodes of +/+ and FOXO3L mice were collected and stained with anti-CD8 and MHC-I tetramers. Bars represent data from 2 independent experiments with 4–7 mice/group/experiment; error bars represent the SEM and * indicates statistical significance at p<.05. (**B**) At 180 days PI, splenocytes were stained with anti-CD44, anti-CD62L, anti-CCR7 and anti-LFA-1 along with anti-CD8 and MHC-I tetramer. Representative plots for CD44, CD62L, CCR7 and LFA-1 are gated on tetramer-binding CD8 T cells. The numbers represent the MFI for the indicated protein. (**C**) Cytokine production by LCMV-specific memory CD8 T cells from +/+ and FOXO3L mice. Representative dot plots (left) are gated on total lymphocytes. The top numbers indicate the MFI for IFNγ while the bottom number in the plot indicates the percentage of total splenocytes that are CD8 and IFNγ positive. Dot plots (right) represent the percentage of IL-2 and/or TNFα producing cells among IFNγ^+ve^ CD8 T cells. The plots represent data from 1 of 2 independent experiments with 4–7 mice/group/experiment. (**D**) +/+ and FOXO3L mice were infected with LCMV and given a BrdU injection at 120 days PI and administered BrdU in drinking water between days 120–128 PI (top). At the end of the BrdU pulse, splenocytes were stained with anti-CD8, MHC-I tetramers and anti-BrdU (top) or anti-Ki67 (bottom). The percentage of BrdU or Ki67 positive cells amongst tetramer positive CD8 T cells was determined by flow cytometry. Bars represent data from at least 5 mice/group.

To determine whether deletion of FOXO3, exclusively from the T cell compartment, would affect CD8 T cell memory generation, FOXO3L and +/+ mice were infected with LCMV and virus-specific memory CD8 T cells were quantified in lymphoid and non-lymphoid tissues at 180 days PI. We observed a significant increase in the number of memory CD8 T cells that are specific to the three immuno-dominant LCMV epitopes (NP396 P<0.02; GP33 P<0.01; GP276 P<0.05) in spleens of FOXO3L mice over the +/+ mice (**Figure 10A**). These data indicated that FOXO3 regulates the magnitude of CD8 T cell memory by T cell intrinsic mechanisms.

Next, we examined whether conditional deficiency of FOXO3 in T cells affected the phenotypic and functional attributes of memory CD8 T cells. The expressions of CD44, CD62L, CCR7 and LFA-1 on +/+ and FOXO3L memory CD8 T cells from spleen, liver and lymph nodes were similar (**Figure 10B**). Additionally, the relative proportions of effector (CD62L^Lo^) and central (CD62L^HI^) memory CD8 T cells were unaffected by FOXO3 deficiency (**Figure 10B**). In response to antigenic stimulation, virus-specific memory CD8 T cells in LCMV-immune FOXO3L−/− mice produced IFNγ, TNFα, and IL-2 at levels comparable to those in +/+ mice (**Figure 10C**). As was the case in our studies of global FOXO3-deficient mice (**Figure 7**), memory CD8 T cells in FOXO3L mice exhibited no difference in homeostatic turnover, as evidenced by BrdU incorporation from day 120 PI mice, pulsed for 8 days and by parallel Ki67 staining (**Figure 10D**). Taken together, data presented in **Figure 10** strongly suggested that FOXO3 regulates the quantity, with no apparent loss in quality, of CD8 T cell memory by T cell intrinsic mechanisms.

## Discussion

The FOXO transcription factors are important regulators of cell cycle progression, apoptosis, and energy metabolism [21], [23], [26], [28], [56]. In the T cell compartment, FOXOs have been implicated in regulating homing of T cells, cytokine receptor expression, and development of regulatory T cells [48], [51], [53], [54]. While it has been reported that FOXO3 may control CD8 T cell expansion, albeit through non T cell intrinsic mechanisms, a role for FOXO3 in memory T cell survival has also been posited [49]. What has not been thoroughly addressed, however, is the T cell intrinsic role of FOXO3 in governing different facets of the physiological polyclonal T cell response to foreign antigens, including the in vivo generation and maintenance of CD8 T cell memory. In the present study, we have systematically examined the T cell-intrinsic role of FOXO3 in controlling the expansion, contraction, and memory phases of the polyclonal CD8 T cell response to an acute viral infection. These studies have provided strong evidence supporting a T cell intrinsic role for FOXO3 in limiting the magnitude of expansion and the number of memory CD8 T cells in a tissue-specific fashion during a physiological response to an acute LCMV infection. These findings have advanced our mechanistic understanding of CD8 T cell homeostasis, and are expected to have implications in the development of effective vaccines.

FOXOs are known to maintain cellular quiescence by mechanisms including the induction of cell cycle inhibitors like p27^KIP1^ and p21^Cip1^ [30], [34], [37], [38]. Downregulation of FOXO activity is believed to be an obligatory step for cell cycle entry in response to mitogenic stimuli [30]. The observed phosphorylation of FOXO1/O3 in LCMV-specific CD8 T cells was readily detectable at day 5 PI but exhibited a sharp decline by day 8 PI. The drop in the phosphorylation in FOXO1/O3 between days 5 and 8 PI coincides with declining viral load and decreased antigenic stimulation. However, we observed a rebound in the phosphorylation of FOXO1/O3 between days 8 and 11 PI. What controls the dynamics of FOXO1/O3 phosphorylation during a CD8 T cell response? In addition to TCR signaling, FOXO1/O3 phosphorylation is regulated by signaling via cytokine receptors such as IL-2, IL-7, and IL-15 [38], [56], [66]. It is possible that FOXO1/O3 is phosphorylated by different extracellular signals at different phases of the T cell response. For example, during the phase of antigen-driven proliferation, IL-7R expression is known to be very low [68], and TCR signaling along with IL-2/IL-15 might drive the phosphorylation of FOXO1/O3. However, after antigen clearance, IL-7 signaling might drive phosphorylation on the surviving IL-7 receptor-expressing MPECs, and eventually their resultant memory cells.

FOXO3 has been reported to suppress expansion of CD8 T cells indirectly by inhibiting IL-6 production by dendritic cells [49]. In this report, however, the T cell intrinsic role of FOXO3 was not assessed in polyclonal CD8 T cells, and monoclonal TCR transgenic CD8 T cells may not always mimic the responses of polyclonal CD8 T cells. In the present study, global FOXO3-deficient mice exhibit increased expansion of polyclonal CD8 T cells specific to multiple epitopes during an acute LCMV infection. Furthermore, infection of T cell-specific conditional FOXO3-deficient mice with LCMV, fully recapitulated the enhanced CD8 T cell expansion seen in global FOXO3−/− mice; even in the absence of CD4 T cells. Therefore, our data implies that FOXO3 suppresses CD8 T cell expansion in vivo by T cell intrinsic and extrinsic mechanisms. This inference is also supported by the reported T cell intrinsic regulation of regulatory T cell development by FOXO1 and FOXO3 [71], [72]. One of the most interesting findings presented in this manuscript is that the effect of FOXO3 deficiency on CD8 T cell expansion and memory is observed in the spleen, but not in the liver or lymph nodes. The enhanced accumulation of effector CD8 T cells preferentially in spleens of FOXO3−/− mice could not be explained by tissue-specific differences in BIM expression in CD8 T cells directly ex vivo; regardless of the tissue (spleen, liver, or lymph nodes), BIM levels in FOXO3−/− CD8 T cells were slightly lower than in +/+ CD8 T cells (**Figure S5**). Additionally, the selective increase in the number of memory CD8 T cells in spleens of FOXO3−/− mice could not be linked to alterations in the expression of molecules such as CD62L, LFA-1, CCR7, and CD44 that regulate T cell trafficking (**Figure 10B**). Tissue-specific effects were observed in both global and T cell-specific conditional FOXO3 knockout mice, which suggests that the local immunological milieu influences the effects of FOXO3 in T cells. It is possible that FOXO3-deficient T cells are hyper-responsive to cellular and environmental cues unique to the spleen during or after cessation of antigen-driven proliferation. Tissue-specific alterations in CD8 T cell homeostasis are not unique to FOXO3 deficiency because lymph node-specific effects on CD8 T cell numbers have been reported in mice deficient for Fas and BIM [64]. Future experiments will address the mechanisms underlying the tissue-specific effects of FOXO3 in regulating CD8 T cell homeostasis.

FOXO3 is known to regulate both proliferation and apoptosis by controlling the transcription of genes like p27^Kip1^, p130, BIM, and Fas ligand [26], [34], [35], [63], [64]. Therefore, the observed increase in the accumulation of LCMV-specific CD8 T cells in FOXO3−/− mice could be attributed to altered proliferation and/or apoptosis. Analysis of in vivo proliferation by multiple strategies indicated that FOXO3 deficiency did not alter proliferation rates of LCMV-specific CD8 T cells in vivo. Clonal expansion of CD8 T cells is associated with concomitant proliferation and apoptosis, therefore, cellular accumulation is the result of the proliferation rate exceeding the apoptotic rate. The competing effects of TGF-β and IL-15 are known to dictate the apoptotic rate of proliferating CD8 T cells, but the signaling mechanisms involved are not well defined [61]. We theorized that IL-7/IL-15 deprivation during antigen-driven proliferation might diminish FOXO3 phosphorylation, and augment the expression of BIM. We find that at day 6 PI, apoptosis of LCMV-specific CD8 T cells was significantly reduced in spleens of FOXO3−/− mice. Additionally, IL-15 deprivation was indeed associated with higher BIM levels in +/+ CD8 T cells than in FOXO3−/− CD8 T cells, which suggested that proliferating FOXO3−/− CD8 T cells might be less susceptible to cytokine withdrawal-induced apoptosis during the expansion phase of the CD8 T cell response. During the early contraction phase (day 8–11 PI), a substantial number of LCMV-specific CD8 T cells are still in cycle (**Figure 5A**), but during this interval the apoptotic rate presumably exceeds the proliferation rate resulting in a net loss of CD8 T cells. Interestingly, FOXO3 deficiency minimally altered the contraction of LCMV-specific CD8 T cells. These data suggest that mechanisms controlling apoptosis of CD8 T cells during expansion and contraction are likely distinct.

Remarkably, the numbers of memory CD8 T cells in the spleen of both FOXO3−/− and FOXO3L mice were substantially higher than in +/+ mice. The number of memory CD8 T cells is dictated by the magnitude of expansion (clonal burst size) and contraction of effector CD8 T cells [5]. The magnitude of increase in the number of memory CD8 T cells in FOXO3−/− or FOXO3L mice reflects enhanced expansion of MPECs (**Figure 2B**) during the primary CD8 T cell response. Importantly, enhancement in the number of memory CD8 T cells induced by FOXO3 deficiency was not associated with detectable alterations in phenotype or effector function. Memory CD8 T cells in LCMV-immune FOXO3−/− mice exhibit strong recall responses and provide effective immunity against a persistent LCMV infection. Thus, FOXO3 deficiency increased the quantity of CD8 T cell memory without affecting their phenotype or effector functions.

Memory CD8 T cells are maintained by IL-7 and IL-15-driven proliferative renewal and phosphorylation of FOXO3 is an integral component of the signaling circuitry triggered by IL-7/IL-15 signaling [11], [54], [66], [67]. Additionally, we have previously shown that deficiency of the cell cycle inhibitor p27^Kip1^, a target gene for FOXO3, enhances the homeostatic turnover of memory CD8 T cells [43]. Surprisingly, despite the suggested importance of FOXO3 in regulating the homeostasis of memory T cells, FOXO3 deficiency exerted minimal effects on the proliferative renewal of antigen-specific memory CD8 T cells in vivo [48]. Studies of human memory T cells have ascribed a negative regulatory role for FOXO3 in the persistence of memory CD4 T cells and FOXO3 deficiency would be expected to increase the number of effector memory cells [48], [73]. However, FOXO3 deficiency did not affect the relative proportions of central and effector memory CD8 T cells. It is plausible that FOXO3 might regulate the persistence of central/effector memory CD4 T cells, and not CD8 T cells. Alternatively, FOXO3 function may be redundant in maintaining fully differentiated memory CD4 and CD8 T cells.

In conclusion, this manuscript documents that FOXO3 plays a critical role in controlling the clonal burst size and the magnitude of CD8 T cell memory by T cell intrinsic mechanisms. Furthermore, the enhanced number of memory CD8 T cells induced by FOXO3 deficiency, is maintained for extended periods without compromising its quality. These findings have important implications in vaccine development, and suggest that modulation of FOXO3 activity during the expansion phase might be a fruitful strategy to bolster vaccine-induced CD8 T cell memory and protective immunity.

## Materials and Methods

### Mice and viral infection

The generation and characterization of the global FOXO3-deficient (FOXO3−/−) mice on the C57BL/6 (B6) background have been described previously [40]. The control wild type B6 (+/+) mice were either littermates or purchased from the National Cancer Institute (Bethesda, MD). Derivation of mice carrying the floxed FOXO3 alleles has been described elsewhere [69], [70]. Mice carrying the floxed FOXO3 alleles were bred with the CD4-Cre mice at UW-Madison to generate the T cell-specific FOXO3−/− (FOXO3L) mice. Littermate +/+ mice were used as controls with the FOXO3L mice. Mice used in experiments were between the ages of 6–8 weeks and all experiments were performed in accordance with the protocols approved by the University of Wisconsin School of Veterinary Medicine Institutional Animal Care and Use Committee (IACUC). The animal committee mandates that institutions and individuals using animals for research, teaching, and/or testing much acknowledge and accept both legal and ethical responsibility for the animals under their care, as specified in the Animal Welfare Act (AWA) and associated Animal Welfare Regulations (AWRs) and Public Health Service (PHS) Policy. Mice were infected with 2×10^5^ PFU of lymphocytic choriomeningitis virus (LCMV) Armstrong strain by intraperitoneal (IP) injection. Mice that have recovered from an infection with LCMV Armstrong were challenged with LCMV-Clone 13 (2×10^6^ PFU by intravenous injection). Tissue viral titers were quantified by plaque assay with Vero cell monolayers [74].

### General flow cytometry

Single cell suspensions of splenocytes were stained with antibodies for surface markers including CD8, CD44, CD122, CD127, CD62L, CCR7, LFA-1 and KLRG-1 (BD Biosciences, Franklin Lakes NJ, eBIOSCEINCE, San Diego CA or Southern Biotech, Birmingham AL) in conjunction with MHC I tetramers (D^b^) specific for the class I-restricted LCMV epitopes, NP396, GP33, and GP276 as previously described [15]. Cells were fixed in 2% paraformaldehyde (PFA) and acquired in a FACSCalibur or LSR II flow cytometer (BD Biosciences, Franklin Lakes NJ). To quantify intracellular cytokine production, splenocytes were incubated for 5 hours at 37°C with LCMV epitope viral peptides in the presence of Brefeldin A. After stimulation, cells were first incubated with antibodies for surface markers. Next, cells were permeabilized and stained for intracellular cytokines (IFNγ, IL-2 and TNFα) using the Cytofix/Cytoperm kit (BD Biosciences, Franklin Lakes NJ). The percentages of cytokine-producing cells were quantified by flow cytometry.

### Phospho-specific flow cytometry

Splenocytes were stained for cell surface markers as above. After cell surface staining, cells were fixed and permeabilized using Phosflow lysis and Phosflow PermWash I reagents (BD Biosciences, Franklin Lakes NJ) according to the manufacturer's recommendations. Next, cells were blocked for 30 minutes on ice in blocking buffer (10% normal goat serum in 2%BSA/PBS) and subsequently stained with either phospho-specific antibodies (Cell Signaling Technology, Danvers MA; P-Akt [T308], P-FOXO1/3 [T24,T32], P-mTOR [S2448]) or non-phospho state-specific antibodies (Akt, mTOR, FOXO3, BIM). As negative controls for staining, antibodies were pre-incubated/blocked with their specific antigenic peptide for 1 hr at room temperature before adding on to the cells. Following incubation with antibody or peptide blocked antibody, cells were washed twice and incubated with secondary antibody (Goat anti-Rabbit ALEXA488; Sigma-Aldrich, St. Louis MO) for 40 minutes. Cells were washed and fixed with 2% PFA. The levels of phospho-specific staining were quantified by flow cytometry. Specific levels of staining (Corrected Mean Fluorescence Intensity [MFI]) were calculated using the formula: difference of observed MFI for the phospho-specific protein and peptide-blocked control, divided by the peptide-blocked control.

### Assessment of in vivo proliferation

To assess in vivo proliferation of antigen specific cells, mice were administered an IP injection of 1.5 mg of 5-Bromo-2′-deoxyuridine ([BrdU] MP Biomedicals, Solon OH) followed by exposure to 0.8 mg/ml of BrdU in drinking water for the rest of the pulse period. Splenocytes were stained for surface markers and determination of BrdU positive cells was performed using a BrdU staining kit (BD Biosciences).

### Ki67, Bcl-2 and Granzyme B staining

Splenocytes were stained for surface markers and MHC I tetramers as described above. After surface staining, cells were fixed and permeabilized using FACS Lysing Solution and FACS Permeabilization Solution 2 reagents (BD Biosciences, Franklin Lakes NJ) and subsequently incubated with antibodies against Ki67, Bcl-2 or Granzyme B (BD Biosciences, Franklin Lakes NJ) for 45 minutes at room temperature. Virus-specific CD8 T cells staining positive for Ki67, Bcl-2 or Granzyme B were visualized using a FACSCalibur flow cytometer. Data is expressed as either a percentage of antigen-specific CD8 T cells positive for the respective protein or MFI for the indicated protein.

### Assessment of apoptosis ex vivo

After 6 days PI, splenocytes from +/+ and FOXO3−/− or FOXO3L mice were isolated and stained with anti-CD8 and MHC-I tetramers as described above except no red blood cell lysis was performed. Annexin V staining (BD Biosciences, Franklin Lakes NJ ) was then carried out according to the manufacturer's protocol, with the exception that all staining was performed on ice. The percentage of Annexin V high cells amongst antigen-specific CD8 T cells was determined by flow cytometry and expressed accordingly.

### CD4 depletion studies

Mice were depleted of CD4 T cells through IP administration of 100 µg of the monoclonal antibody GK1.5 (eBioscience, San Diego CA) at days 0 and 4 relative to LCMV infection.

### Western analysis

T cells and non-T cells were purified from spleens of +/+ and FOXO3L mice using the anti-CD90.2 based MACS cell separation system (Miltenyi Biotec, Auburn CA). Purity of cells was >93%. Cells were subsequently lysed in buffer (50 mM HEPES, 100 Mm NaCl, 10 mM EDTA, 10 Mm NaF, 4 Mm Na(PO_4_)_2_, 1% Triton X-100, 5 µg/ml Aprotinin, 1 Mm Phenylmethylsulfonylflouride), sonicated, and total protein levels in each lysate were determined by the Bicinchoninic Acid protein assay. (Sigma-Aldrich, St. Louis, MO). 20 µg samples were loaded and resolved on a 10% SDS-PAGE. Total levels of FOXO3 protein in each sample were detected using a Rabbit primary antibody specific for FOXO3 (Cell Signaling Technology, Danvers MA) followed by a Donkey anti Rabbit F(ab)_2_ fragment HRP-conjugated secondary antibody (Thermo Fisher, Rockford IL) Bands were visualized using chemiluminescence reagents (Thermo Fisher, Rockford IL) and presented by use of an HP Deskscan system (Hewlett-Packard, Palo Alto CA). Blots initially probed for FOXO3 were subsequently stripped and re-probed with β-Actin (Sigma-Aldrich, St. Louis MO) to serve as a loading control.

## Supporting Information

Figure S1 **Early investigation of proliferation in +/+ and FOXO3−/− mice during LCMV infection.** After 5 days of infection with LCMV, splenocytes were isolated from +/+ and FOXO3−/− mice and stained with anti-CD8, MHC-I tetramer and anti-Ki67. The percentage of Ki67 positive cells amongst tetramer binding CD8 T cells was determined by flow cytometry. Data is the mean from at least 2 individual experiments with 4–6 mice/group/experiment.
(TIF)

Figure S2 **Representative plots of BIM and Bcl-2 MFIs from day 6 PI in +/+ and FOXO3−/− mice after 6 hours of culture with and without IL-15.** Splenocytes from +/+ and FOXO3−/− mice were isolated after 6 days PI. Splenocytes were cultured for 6 hours with and without IL-15 (10 ng/ml). After 6 hours, splenocytes were stained with anti-CD8, MHC-I tetramers and anti-BIM, anti-Bcl-2 or the respective isotype control. Representative tracings of BIM (top) or the isotype control from pooled samples without (left) or with 10 ng/ml IL-15 (right) are shown. Representative Bcl-2 or isotype control MFIs (bottom) in +/+ and FOXO3−/− mice without (left) or with 10 ng/ml IL-15 (right) are also illustrated. Data are from pooled samples from at least two experiments.
(TIF)

Figure S3 **Characterization of FOXO3−/− and FOXO3L mice.** (**A**) Mononuclear cells from spleens of +/+, FOXO3+/− and FOXO3−/− mice were isolated and stained with anti-CD8, MHC-I tetramer, anti-FOXO3 antibody or FOXO3 antibody pre-incubated with its specific antigenic peptide. Histograms represent the MFI of FOXO3 signal for each FOXO3 variant. Numbers represent MFI of FOXO3 for each group. (**B**) Mononuclear cells from spleens of +/+ and FOXO3L mice were stained for anti-CD8, anti-CD4, anti-B220, anti CD11c and anti-FOXO3. Histograms represent the MFI of the FOXO3 signal gated on either total positive CD8s, CD4s, B220 or CD11c as indicated. Numbers represent MFI of FOXO3 for each group. (**C**) Splenocytes from naïve +/+ and FOXO3L mice were collected and T cell and non-T cell fractions were purified using the MACS system (Miltenyi Biotec, Auburn CA) by positive selection for CD90.2. SDS-PAGE followed by immuno-blotting with anti-FOXO3 shows preferential loss of FOXO3 in the T cell compartment of FOXO3L mice but not +/+ mice. β-Actin probing was used to ensure equal loading. (**D**) After 8 days of LCMV infection, mononuclear cells from spleens of +/+, FOXO3−/− and FOXO3L mice were isolated and stained with anti-CD8, MHC-I tetramer, and anti-FOXO3 antibody. Histograms represent the FOXO3 signal from GP33^+ve^ CD8 T cells for each group. Numbers are the MFI of FOXO3 for each respective group.
(TIF)

Figure S4 **Investigation of proliferation during early infection in +/+ and FOXO3L mice.** At 5 days PI, lymphocytes from spleens of +/+ and FOXO3L mice were collected and stained with anti-CD8, MHC-I tetramers and anti-Ki67. The percentage of Ki67^+ve^ cells amongst tetramer binding CD8 T cells was determined by flow cytometry. Data is the mean from at least 2 individual experiments with 3–6 mice/group/experiment.
(TIF)

Figure S5 **Total levels of BIM and Bcl-2, measured directly ex-vivo at 6 days PI.** Total levels of immuno-reactive BIM (left) or Bcl-2 (right) in tetramer positive CD8 T cells from spleen, liver and lymph nodes were assessed directly ex-vivo on day 6 PI. Each solid bar represents the observed MFI for BIM or Bcl-2, and each checkered bar represents the MFI for the respective isotype control.
(TIF)

## References

[1] Jameson SC, Masopust D (2009). Diversity in T cell memory: An embarrassment of riches.. Immunity.

[2] Pulendran B, Ahmed R (2011). Immunological mechanisms of vaccination.. Nat Immunol.

[3] Pulendran B, Ahmed R (2006). Translating innate immunity into immunological memory: Implications for vaccine development.. Cell.

[4] Sallusto F, Lanzavecchia A, Araki K, Ahmed R (2010). From vaccines to memory and back.. Immunity.

[5] Ahmed R, Gray D (1996). Immunological memory and protective immunity: Understanding their relation.. Science.

[6] Kaech SM, Ahmed R (2003). Immunology. CD8 T cells remember with a little help.. Science.

[7] Kalia V, Sarkar S, Ahmed R (2010). CD8 T-cell memory differentiation during acute and chronic viral infections.. Adv Exp Med Biol.

[8] Sprent J, Surh CD (2011). Normal T cell homeostasis: The conversion of naive cells into memory-phenotype cells.. Nat Immunol.

[9] Barber GN (2001). Host defense, viruses and apoptosis.. Cell Death Differ.

[10] Gourley TS, Wherry EJ, Masopust D, Ahmed R (2004). Generation and maintenance of immunological memory.. Semin Immunol.

[11] Hand TW, Morre M, Kaech SM (2007). Expression of IL-7 receptor alpha is necessary but not sufficient for the formation of memory CD8 T cells during viral infection.. Proc Natl Acad Sci U S A.

[12] Masopust D, Murali-Krishna K, Ahmed R (2007). Quantitating the magnitude of the lymphocytic choriomeningitis virus-specific CD8 T-cell response: It is even bigger than we thought.. J Virol.

[13] Hand TW, Kaech SM (2009). Intrinsic and extrinsic control of effector T cell survival and memory T cell development.. Immunol Res.

[14] Haring JS, Badovinac VP, Harty JT (2006). Inflaming the CD8+ T cell response.. Immunity.

[15] Murali-Krishna K, Altman JD, Suresh M, Sourdive DJD, Zajac AJ (1998). Counting antigen-specific CD8 T cells: A reevaluation of bystander activation during viral infection.. Immunity.

[16] Joshi NS, Cui W, Chandele A, Lee HK, Urso DR (2007). Inflammation directs memory precursor and short-lived effector CD8+ T cell fates via the graded expression of T-bet transcription factor.. Immunity.

[17] Sarkar S, Kalia V, Haining WN, Konieczny BT, Subramaniam S (2008). Functional and genomic profiling of effector CD8 T cell subsets with distinct memory fates.. J Exp Med.

[18] Burgering BM, Kops GJ (2002). Cell cycle and death control: Long live forkheads.. Trends Biochem Sci.

[19] Castrillon DH, Miao L, Kollipara R, Horner JW, DePinho RA (2003). Suppression of ovarian follicle activation in mice by the transcription factor Foxo3a.. Science.

[20] Neufeld TP (2003). Shrinkage control: Regulation of insulin-mediated growth by FOXO transcription factors.. J Biol.

[21] Accili D, Arden KC (2004). FoxOs at the crossroads of cellular metabolism, differentiation, and transformation.. Cell.

[22] Hosaka T, Biggs WH, Tieu D, Boyer AD, Varki NM (2004). Disruption of forkhead transcription factor (FOXO) family members in mice reveals their functional diversification.. Proc Natl Acad Sci U S A.

[23] Barthel A, Schmoll D, Unterman TG (2005). FoxO proteins in insulin action and metabolism.. Trends Endocrinol Metab.

[24] Peng SL (2008). Foxo in the immune system.. Oncogene.

[25] van der Vos KE, Coffer PJ (2008). FOXO-binding partners: It takes two to tango.. Oncogene.

[26] van der Vos KE, Coffer PJ (2011). The extending network of FOXO transcriptional target genes.. Antioxid Redox Signal.

[27] Jacobs FM, van der Heide LP, Wijchers PJ, Burbach JP, Hoekman MF (2003). FoxO6, a novel member of the FoxO class of transcription factors with distinct shuttling dynamics.. J Biol Chem.

[28] Calnan DR, Brunet A (2008). The FoxO code.. Oncogene.

[29] Biggs WH, Meisenhelder J, Hunter T, Cavenee WK, Arden KC (1999). Protein kinase B/Akt-mediated phosphorylation promotes nuclear exclusion of the winged helix transcription factor FKHR1.. Proc Natl Acad Sci U S A.

[30] Kops GJPL, Medema RH, Glassford J, Essers MAG, Dijkers PF (2002). Control of cell cycle exit and entry by protein kinase B-regulated forkhead transcription factors.. Mol Cell Biol.

[31] Van Der Heide LP, Hoekman MF, Smidt MP (2004). The ins and outs of FoxO shuttling: Mechanisms of FoxO translocation and transcriptional regulation.. Biochem J.

[32] Strasser A, Harris AW, Huang DC, Krammer PH, Cory S (1995). Bcl-2 and Fas/APO-1 regulate distinct pathways to lymphocyte apoptosis.. EMBO J.

[33] Bouillet P, Metcalf D, Huang DCS, Tarlinton DM, Kay TWH (1999). Proapoptotic bcl-2 relative bim required for certain apoptotic responses, leukocyte homeostasis, and to preclude autoimmunity.. Science.

[34] Dijkers PF, Medema RH, Lammers JW, Koenderman L, Coffer PJ (2000). Expression of the pro-apoptotic bcl-2 family member bim is regulated by the forkhead transcription factor FKHR-L1.. Curr Biol.

[35] Dijkers PF, Medema RH, Pals C, Banerji L, Thomas NSB (2000). Forkhead transcription factor FKHR-L1 modulates cytokine-dependent transcriptional regulation of p27KIP1.. Mol Cell Biol.

[36] Grayson JM, Zajac AJ, Altman JD, Ahmed R (2000). Cutting edge: Increased expression of bcl-2 in antigen-specific memory CD8+ T cells.. J Immunol.

[37] Sharpless NE, DePinho RA (2002). P53: Good Cop/bad cop.. Cell.

[38] Stahl M, Dijkers PF, Kops GJ, Lens SM, Coffer PJ (2002). The forkhead transcription factor FoxO regulates transcription of p27Kip1 and bim in response to IL-2.. J Immunol.

[39] Wan J, Martinvalet D, Ji X, Lois C, Kaech SM (2003). The bcl-2 family pro-apoptotic molecule, BNIP3 regulates activation-induced cell death of effector cytotoxic T lymphocytes.. Immunology.

[40] Lin L, Hron JD, Peng SL (2004). Regulation of NF-kappaB, th activation, and autoinflammation by the forkhead transcription factor Foxo3a.. Immunity.

[41] Tait ED, Hunter CA (2009). The foxo and the hound: Chasing the in vivo regulation of T cell populations during infection.. Nat Immunol.

[42] Zhou W, Cao Q, Peng Y, Zhang QJ, Castrillon DH (2009). FoxO4 inhibits NF-kappaB and protects mice against colonic injury and inflammation.. Gastroenterology.

[43] Singh A, Jatzek A, Plisch EH, Srinivasan R, Svaren J (2010). Regulation of memory CD8 T-cell differentiation by cyclin-dependent kinase inhibitor p27Kip1.. Mol Cell Biol.

[44] Dijkers PF, Birkenkamp KU, Lam EW, Thomas NS, Lammers JW (2002). FKHR-L1 can act as a critical effector of cell death induced by cytokine withdrawal.. J Cell Biol.

[45] Martinez-Gac L, Alvarez B, Garcia Z, Marques M, Arrizabalaga M (2004). Phosphoinositide 3-kinase and forkhead, a switch for cell division.. Biochem Soc Trans.

[46] Birkenkamp KU, Coffer PJ (2003). FOXO transcription factors as regulators of immune homeostasis: Molecules to die for?. J Immunol.

[47] Peng SL (2007). Immune regulation by foxo transcription factors.. Autoimmunity.

[48] Riou C, Yassine-Diab B, Van grevenynghe J, Somogyi R, Greller LD (2007). Convergence of TCR and cytokine signaling leads to FOXO3a phosphorylation and drives the survival of CD4+ central memory T cells.. J Exp Med.

[49] Dejean AS, Beisner DR, Ch'en IL, Kerdiles YM, Babour A (2009). Transcription factor Foxo3 controls the magnitude of T cell immune responses by modulating the function of dendritic cells.. Nat Immunol.

[50] Hedrick SM (2009). The cunning little vixen: Foxo and the cycle of life and death.. Nat Immunol.

[51] Ohkura N, Sakaguchi S (2010). Foxo1 and Foxo3 help Foxp3.. Immunity.

[52] Dejean AS, Hedrick SM, Kerdiles YM (2011). Highly specialized role of forkhead box O transcription factors in the immune system.. Antioxid Redox Signal.

[53] Ouyang W, Li MO (2011). Foxo: In command of T lymphocyte homeostasis and tolerance.. Trends Immunol.

[54] Kerdiles YM, Beisner DR, Tinoco R, Dejean AS, Castrillon DH (2009). Foxo1 links homing and survival of naive T cells by regulating L-selectin, CCR7 and interleukin 7 receptor.. Nat Immunol.

[55] Coffer PJ, Burgering BM (2004). Forkhead-box transcription factors and their role in the immune system.. Nat Rev Immunol.

[56] Barata JT, Silva A, Brandao JG, Nadler LM, Cardoso AA (2004). Activation of PI3K is indispensable for interleukin 7-mediated viability, proliferation, glucose use, and growth of T cell acute lymphoblastic leukemia cells.. J Exp Med.

[57] Finlay D, Cantrell D (2010). Phosphoinositide 3-kinase and the mammalian target of rapamycin pathways control T cell migration.. Ann N Y Acad Sci.

[58] Suresh M, Lanier G, Large MK, Whitmire JK, Altman JD (2002). Role of lymphotoxin alpha in T-cell responses during an acute viral infection.. J Virol.

[59] Wherry EJ, Teichgraber V, Becker TC, Masopust D, Kaech SM (2003). Lineage relationship and protective immunity of memory CD8 T cell subsets.. Nat Immunol.

[60] Hand TW, Cui W, Jung YW, Sefik E, Joshi NS (2010). Differential effects of STAT5 and PI3K/AKT signaling on effector and memory CD8 T-cell survival.. Proc Natl Acad Sci U S A.

[61] Sanjabi S, Mosaheb MM, Flavell RA (2009). Opposing effects of TGF-β and IL-15 cytokines control the number of short-lived effector CD8+ T cells.. Immunity.

[62] Ahmed MB, Belhadj Hmida N, Moes N, Buyse S, Abdeladhim M (2009). IL-15 renders conventional lymphocytes resistant to suppressive functions of regulatory T cells through activation of the phosphatidylinositol 3-kinase pathway.. J Immunol.

[63] Wojciechowski S, Tripathi P, Bourdeau T, Acero L, Grimes HL (2007). Bim/Bcl-2 balance is critical for maintaining naive and memory T cell homeostasis.. J Exp Med.

[64] Weant AE, Michalek RD, Khan IU, Holbrook BC, Willingham MC (2008). Apoptosis regulators bim and fas function concurrently to control autoimmunity and CD8+ T cell contraction.. Immunity.

[65] Kurtulus S, Tripathi P, Moreno-Fernandez ME, Sholl A, Katz JD (2011). Bcl-2 allows effector and memory CD8+ T cells to tolerate higher expression of bim.. J Immunol.

[66] Zhang X, Sun S, Hwang I, Tough DF, Sprent J (1998). Potent and selective stimulation of memory-phenotype CD8+ T cells in vivo by IL-15.. Immunity.

[67] Becker TC, Wherry EJ, Boone D, Murali-Krishna K, Antia R (2002). Interleukin 15 is required for proliferative renewal of virus-specific memory CD8 T cells.. J Exp Med.

[68] Kaech SM, Tan JT, Wherry EJ, Konieczny BT, Surh CD (2003). Selective expression of the interleukin 7 receptor identifies effector CD8 T cells that give rise to long-lived memory cells.. Nat Immunol.

[69] Paik J, Kollipara R, Chu G, Ji H, Xiao Y (2007). FoxOs are lineage-restricted redundant tumor suppressors and regulate endothelial cell homeostasis.. Cell.

[70] Tothova Z, Kollipara R, Huntly BJ, Lee BH, Castrillon DH (2007). FoxOs are critical mediators of hematopoietic stem cell resistance to physiologic oxidative stress.. Cell.

[71] Harada Y, Harada Y, Elly C, Ying G, Paik JH (2010). Transcription factors Foxo3a and Foxo1 couple the E3 ligase cbl-b to the induction of Foxp3 expression in induced regulatory T cells.. J Exp Med.

[72] Ouyang W, Beckett O, Ma Q, Paik JH, DePinho RA (2010). Foxo proteins cooperatively control the differentiation of Foxp3+ regulatory T cells.. Nat Immunol.

[73] van Grevenynghe J, Procopio FA, He Z, Chomont N, Riou C (2008). Transcription factor FOXO3a controls the persistence of memory CD4(+) T cells during HIV infection.. Nat Med.

[74] Ahmed R, Salmi A, Butler LD, Chiller JM, Oldstone MB (1984). Selection of genetic variants of lymphocytic choriomeningitis virus in spleens of persistently infected mice. role in suppression of cytotoxic T lymphocyte response and viral persistence.. J Exp Med.

