# Supplementary figures and images for: FOXO3 Regulates CD8 T Cell Memory by T Cell-Intrinsic Mechanisms
Source: PLoS Pathog. 2012 Feb 16;8(2):e1002533. doi: 10.1371/journal.ppat.1002533 (PMC3280979; doi:10.1371/journal.ppat.1002533)

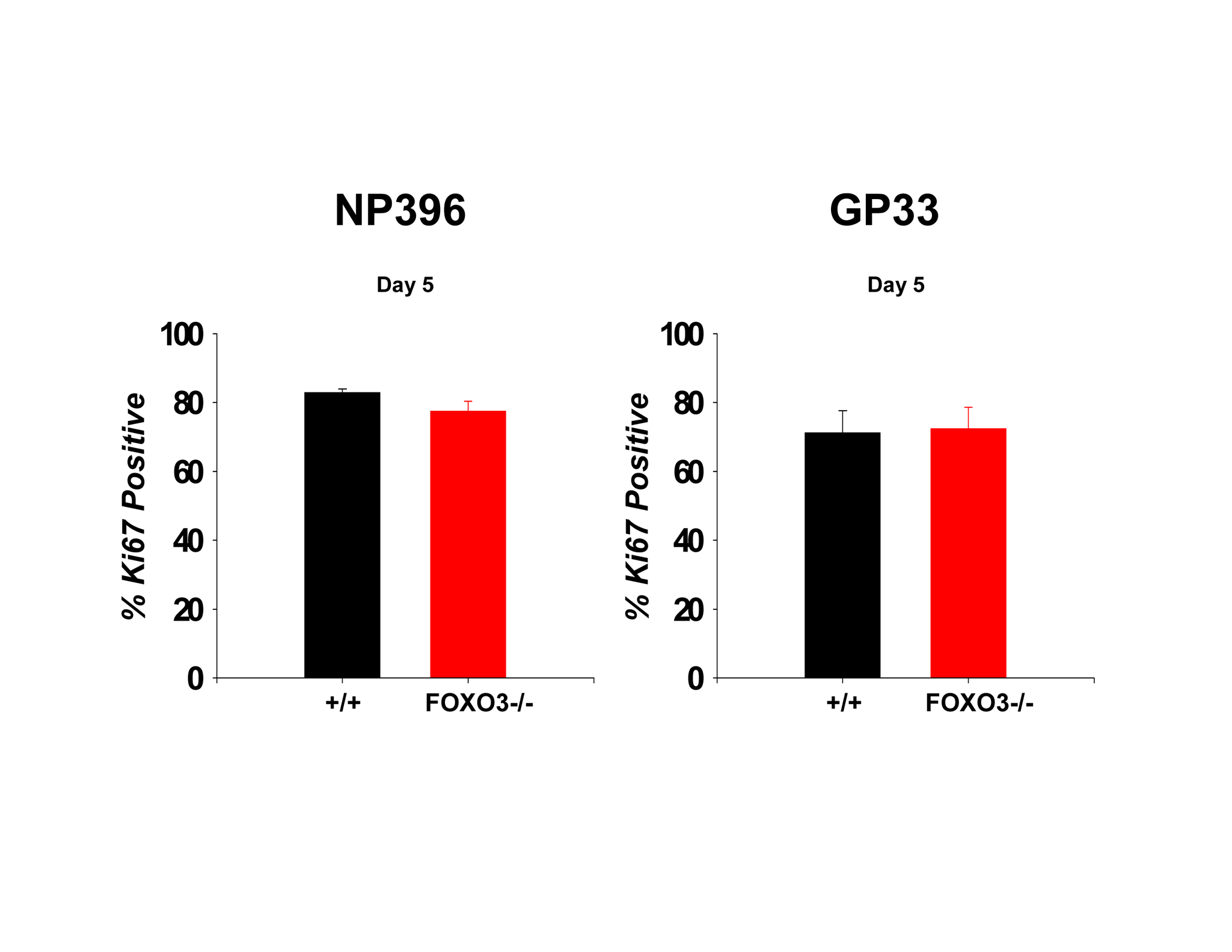

Supplement: Figure S1 — Early investigation of proliferation in +/+ and FOXO3−/− mice during LCMV infection. After 5 days of infection with LCMV, splenocytes were isolated from +/+ and FOXO3−/− mice and stained with anti-CD8, MHC-I tetramer and anti-Ki67. The percentage of Ki67 positive cells amongst tetramer binding CD8 T cells was determined by flow cytometry. Data is the mean from at least 2 individual experiments with 4–6 mice/group/experiment. (TIF) [file ppat.1002533.s001.tif]

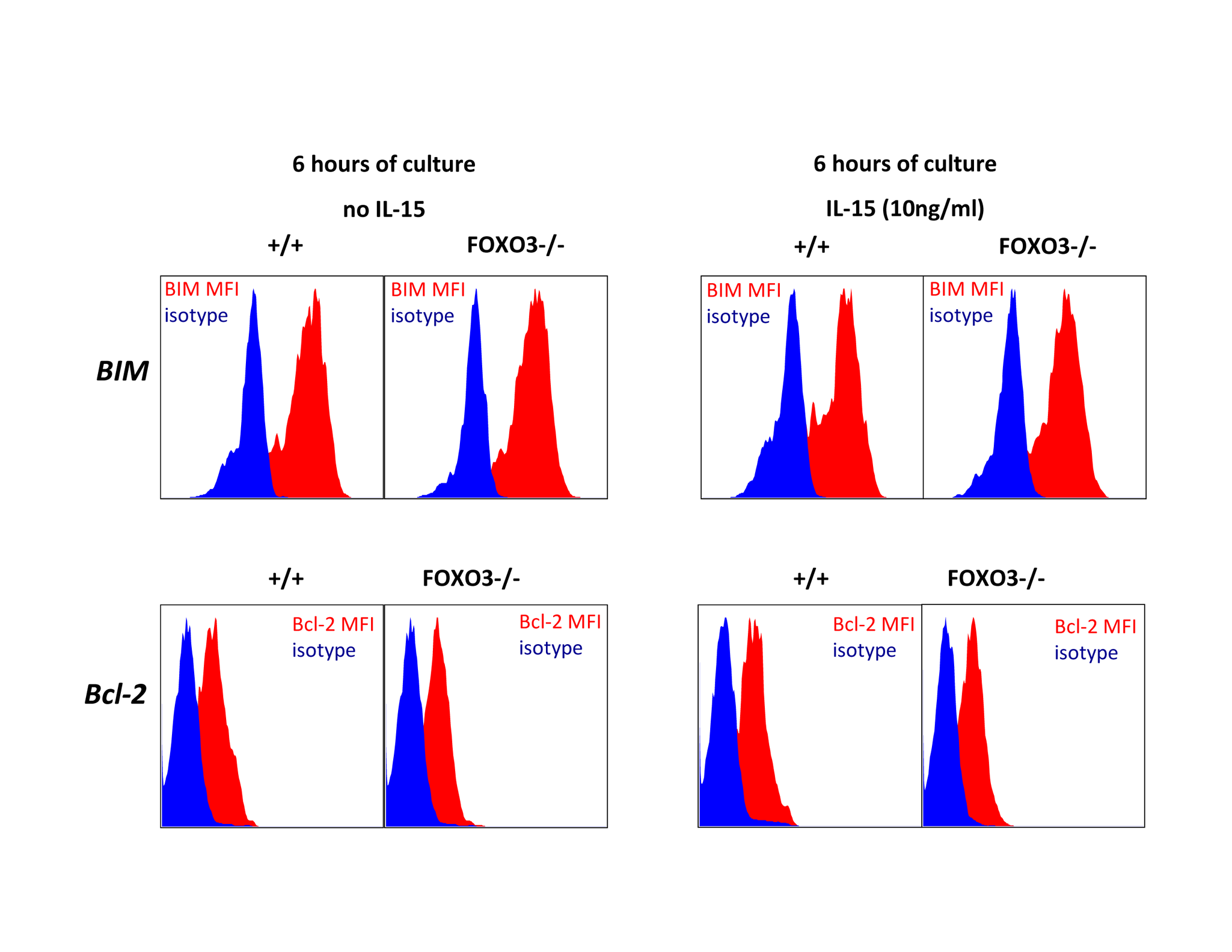

Supplement: Figure S2 — Representative plots of BIM and Bcl-2 MFIs from day 6 PI in +/+ and FOXO3−/− mice after 6 hours of culture with and without IL-15. Splenocytes from +/+ and FOXO3−/− mice were isolated after 6 days PI. Splenocytes were cultured for 6 hours with and without IL-15 (10 ng/ml). After 6 hours, splenocytes were stained with anti-CD8, MHC-I tetramers and anti-BIM, anti-Bcl-2 or the respective isotype control. Representative tracings of BIM (top) or the isotype control from pooled samples without (left) or with 10 ng/ml IL-15 (right) are shown. Representative Bcl-2 or isotype control MFIs (bottom) in +/+ and FOXO3−/− mice without (left) or with 10 ng/ml IL-15 (right) are also illustrated. Data are from pooled samples from at least two experiments. (TIF) [file ppat.1002533.s002.tif]

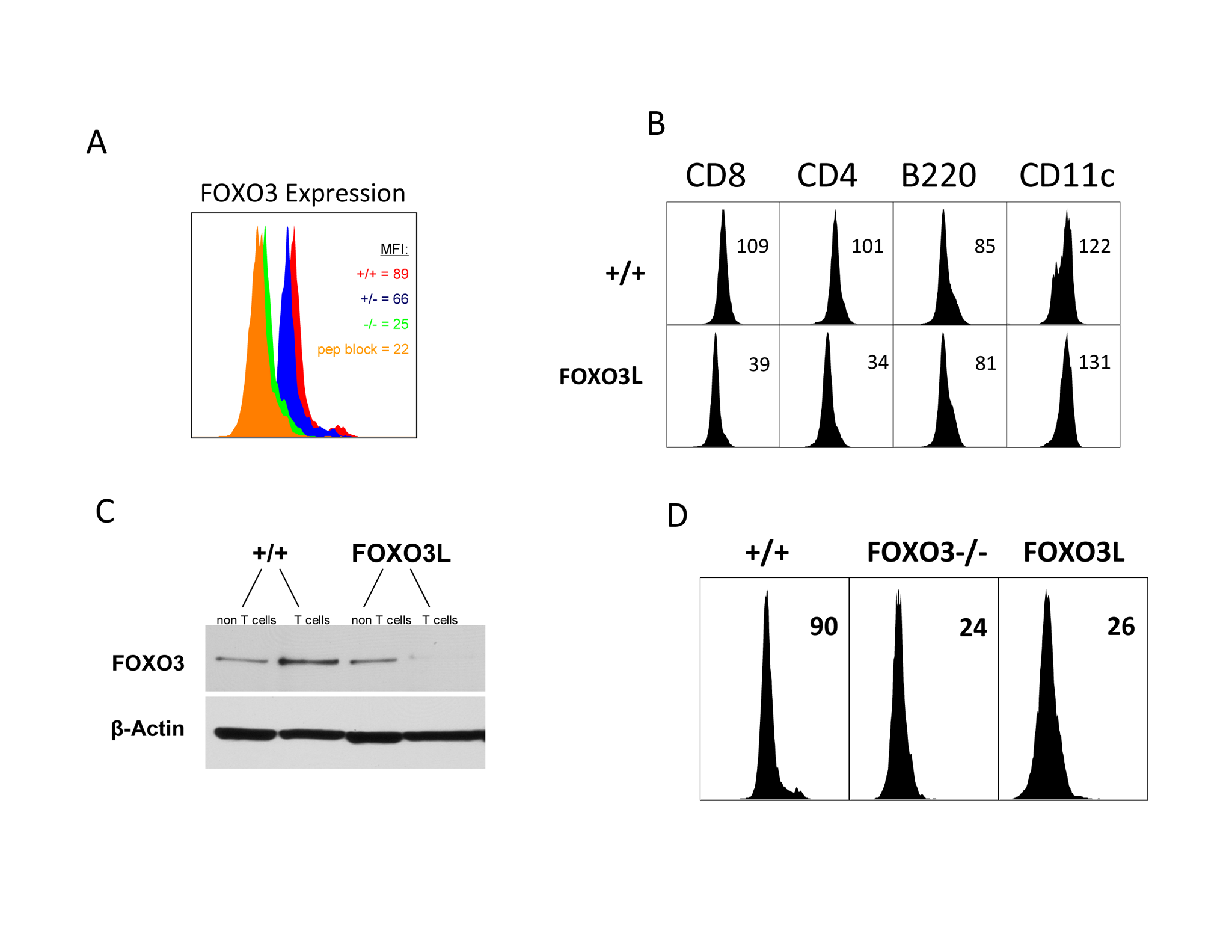

Supplement: Figure S3 — Characterization of FOXO3−/− and FOXO3L mice. (A) Mononuclear cells from spleens of +/+, FOXO3+/− and FOXO3−/− mice were isolated and stained with anti-CD8, MHC-I tetramer, anti-FOXO3 antibody or FOXO3 antibody pre-incubated with its specific antigenic peptide. Histograms represent the MFI of FOXO3 signal for each FOXO3 variant. Numbers represent MFI of FOXO3 for each group. (B) Mononuclear cells from spleens of +/+ and FOXO3L mice were stained for anti-CD8, anti-CD4, anti-B220, anti CD11c and anti-FOXO3. Histograms represent the MFI of the FOXO3 signal gated on either total positive CD8s, CD4s, B220 or CD11c as indicated. Numbers represent MFI of FOXO3 for each group. (C) Splenocytes from naïve +/+ and FOXO3L mice were collected and T cell and non-T cell fractions were purified using the MACS system (Miltenyi Biotec, Auburn CA) by positive selection for CD90.2. SDS-PAGE followed by immuno-blotting with anti-FOXO3 shows preferential loss of FOXO3 in the T cell compartment of FOXO3L mice but not +/+ mice. β-Actin probing was used to ensure equal loading. (D) After 8 days of LCMV infection, mononuclear cells from spleens of +/+, FOXO3−/− and FOXO3L mice were isolated and stained with anti-CD8, MHC-I tetramer, and anti-FOXO3 antibody. Histograms represent the FOXO3 signal from GP33+ve CD8 T cells for each group. Numbers are the MFI of FOXO3 for each respective group. (TIF) [file ppat.1002533.s003.tif]

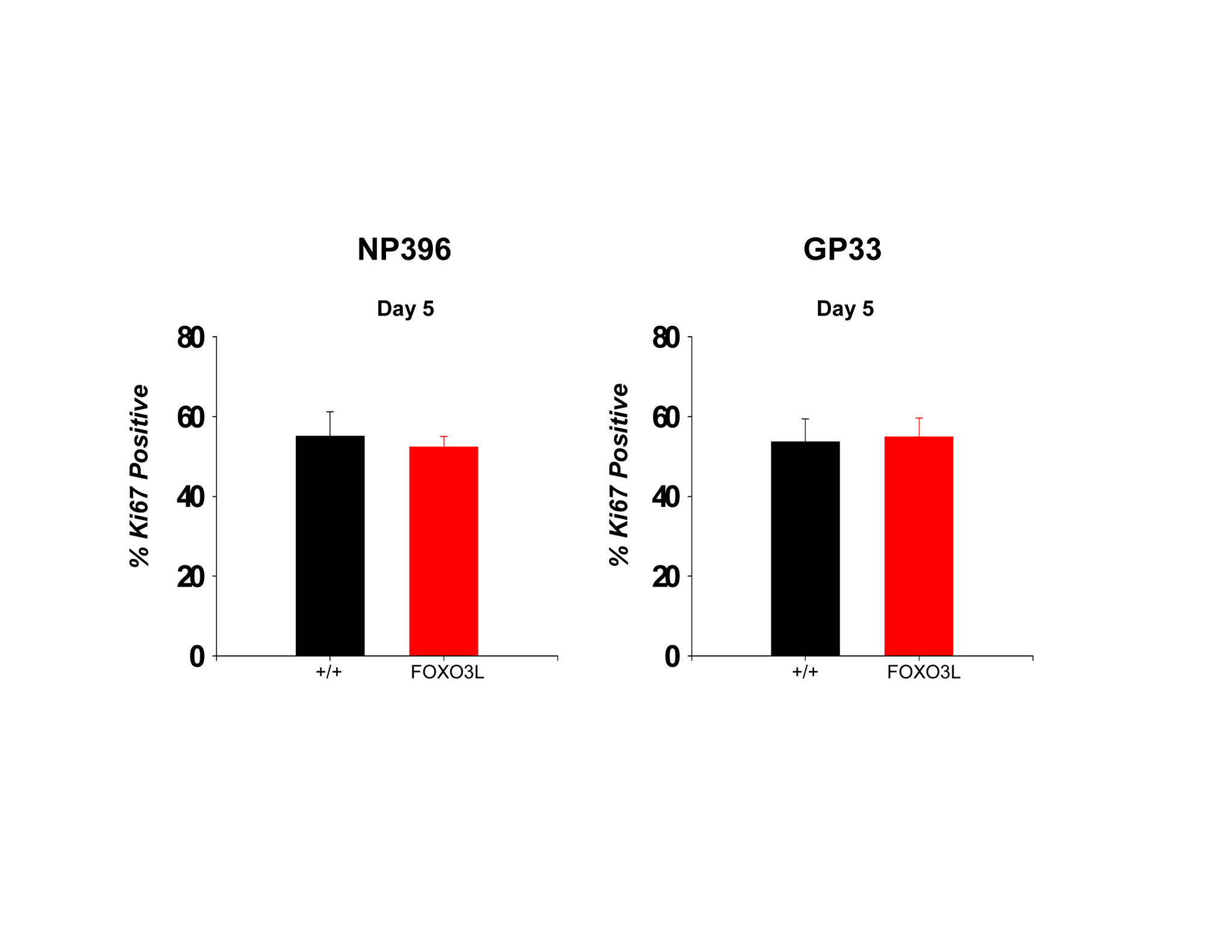

Supplement: Figure S4 — Investigation of proliferation during early infection in +/+ and FOXO3L mice. At 5 days PI, lymphocytes from spleens of +/+ and FOXO3L mice were collected and stained with anti-CD8, MHC-I tetramers and anti-Ki67. The percentage of Ki67+ve cells amongst tetramer binding CD8 T cells was determined by flow cytometry. Data is the mean from at least 2 individual experiments with 3–6 mice/group/experiment. (TIF) [file ppat.1002533.s004.tif]

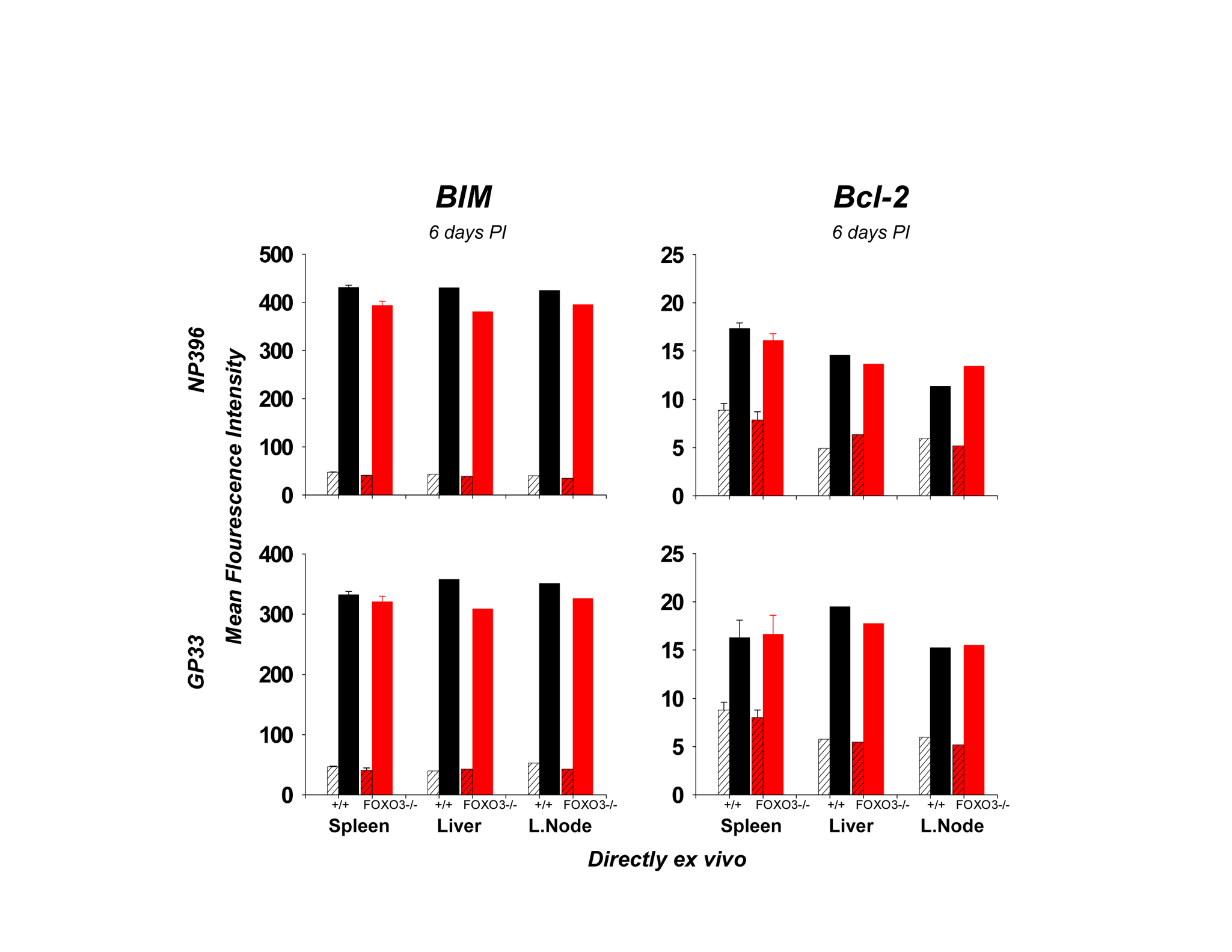

Supplement: Figure S5 — Total levels of BIM and Bcl-2, measured directly ex-vivo at 6 days PI. Total levels of immuno-reactive BIM (left) or Bcl-2 (right) in tetramer positive CD8 T cells from spleen, liver and lymph nodes were assessed directly ex-vivo on day 6 PI. Each solid bar represents the observed MFI for BIM or Bcl-2, and each checkered bar represents the MFI for the respective isotype control. (TIF) [file ppat.1002533.s005.tif]
